# Whole-brain mapping of basal forebrain cholinergic neurons reveals a long-range reciprocal input-output loop between distinct subtypes

**DOI:** 10.1126/sciadv.adt1617

**Published:** 2025-05-30

**Authors:** Zhaonan Chen, Yanmei Liu, Yunqi Yang, Lizhao Wang, Meiling Qin, Zhishan Jiang, Min Xu, Siyu Zhang

**Affiliations:** ^1^ Department of Ophthalmology, Songjiang Hospital and Songjiang Research Institute, Shanghai Key Laboratory of Emotions and Affective Disorders, Shanghai Jiao Tong University School of Medicine, Shanghai 201600, China.; ^2^ Department of Anatomy and Physiology, Shanghai Jiao Tong University School of Medicine, Shanghai 200025, China.; ^3^ Institute of Neuroscience, CAS Center for Excellence in Brain Science and Intelligence Technology, State Key Laboratory of Neuroscience, Chinese Academy of Sciences, Shanghai 200031, China.

## Abstract

Basal forebrain cholinergic neurons (BFCNs) influence cognition and emotion through specific acetylcholine release in various brain regions, including the prefrontal cortices and basolateral amygdala (BLA). Acetylcholine release is controlled by distinct BFCN subtypes, modulated by excitatory and inhibitory inputs. However, the organization of the whole-brain input-output networks of these subtypes remains unclear. Here, we identified two distinct BFCN subtypes—BFCN_→ACA_ and BFCN_→BLA_—innervating the anterior cingulate cortex (ACA) and BLA, each with unique distributions, electrophysiological properties, and projection patterns. Combining rabies-virus–assisted mapping and triple-plex RNAscope hybridization, we characterized their whole-brain input networks, identifying unique excitatory and shared inhibitory inputs for these subtypes. Moreover, our results reveal a long-range reciprocal input-output loop: BFCN_→ACA_ neurons target the isocortex, their shared excitatory-input source, whereas BFCN_→BLA_ neurons target shared inhibitory-input sources such as the striatum and pallidum, thus enabling dynamic interactions among these BFCN subtypes. Our study deepens understanding of cholinergic modulation in cognition and emotion and provides insights into their functional interactions.

## Teaser

A long-range loop between BFCN subtypes contributes to interaction between cholinergic modulation of cognition and emotion.

## INTRODUCTION

Basal forebrain cholinergic neurons (BFCNs) affect cognition and emotion through highly dynamic and selective acetylcholine (ACh) release across various brain regions, including the prefrontal cortices and the basolateral amygdala (BLA) (*1*–*6*). Elevated ACh levels in the prefrontal cortices enhance top-down modulation on sensory cortices, driving attention toward salient sensory stimuli and facilitating cue detection (*2*, *7*–*10*). In addition, ACh release in the BLA, responding to both negative and positive valence stimuli (punishment and reward), contributes to the encoding of emotionally salient memories (*11*–*13*). Dysfunction of BFCNs, which has been implicated in conditions like Alzheimer’s disease, is closely associated with both cognitive and emotional impairment (*14*–*20*).

Each BFCN targets multiple downstream areas, and BFCNs that share common targets are organized into distinct yet overlapping “pools” (*1*, *21*–*23*). However, anatomically defined basal forebrain (BF) subregions often project to multiple, sometimes overlapping, target areas (*1*, *5*). For instance, BFCNs projecting to the prefrontal cortices and those targeting the BLA are extensively rostro-caudally intermingled along much of the BF (*24*).

Previous studies have mapped the inputs to BFCNs, identifying input sources such as the striatum (STR), hypothalamus (HY), and amygdala (*25*, *26*). More recent studies focusing on subgroups of BFCNs projecting to various cortical areas and the amygdala have revealed functionally organized input-output patterns, such as amygdala-projecting BFCNs receiving more inputs from subregions of the amygdala (*24*, *27*). It is well-established that most neurons of the STR are inhibitory (GABAergic), whereas the HY and amygdala contain both excitatory (glutamatergic) and inhibitory neurons (*28*–*30*), yet the specific types of these input neurons remain ambiguous.

Cognition and emotion interact dynamically, for example, in threat detection and avoidance, during which fear biases attention toward potentially threatening stimuli (*31*–*33*). While BFCNs affect both cognition and emotion, how their interactions are coordinated remains largely unexplored. Our research group has a long-standing interest in the top-down modulation of visual processing (*34*–*38*), and the anterior cingulate cortex (ACA) is a prefrontal cortical area involved in top-down visual attention (*36*, *38*–*41*). Previous studies have speculated that BFCNs innervating the prefrontal cortices are less likely to overlap with those innervating the BLA (*5*, *24*). Therefore, investigating the interactions among the input and output networks of BFCNs targeting the ACA and the BLA may yield a deeper understanding of the interactions between cholinergic modulation of attention and emotion.

Here, we found that the BFCNs innervating the ACA and the BLA are two distinct subtypes: BFCN_→ACA_ and BFCN_→BLA_. Each subtype is characterized by unique distribution patterns, electrophysiological properties, and whole-brain projection patterns. BFCN_→ACA_ neurons innervate the isocortex, whereas BFCN_→BLA_ neurons target the cortical subplate (CTXsp), pallidum (PAL), and STR. Using rabies virus (RV)–assisted mapping and triple-plex RNAscope in situ hybridization, we distinguished their excitatory and inhibitory inputs, revealing distinct excitatory-input sources: BFCN_→ACA_ neurons receive excitatory inputs from the isocortex, midbrain (MB), and HY; BFCN_→BLA_ neurons are targeted by the isocortex, CTXsp, and thalamus (TH). Despite these differences in excitatory-input sources, both subtypes receive inhibitory inputs from the STR, PAL, and HY. We also identified a long-range reciprocal input-output loop between BFCN_→ACA_ and BFCN_→BLA_ neurons: BFCN_→ACA_ neurons target a shared excitatory-input source―the isocortex―whereas BFCN_→BLA_ neurons project to shared inhibitory-input sources, including the STR and PAL. These insights into the organization of BF cholinergic input-output networks advance our understanding of cholinergic modulation in cognition and emotion.

## RESULTS

### BFCN_→ACA_ and BFCN_→BLA_ are distinct subtypes with unique distribution patterns

**Fig. 1.**
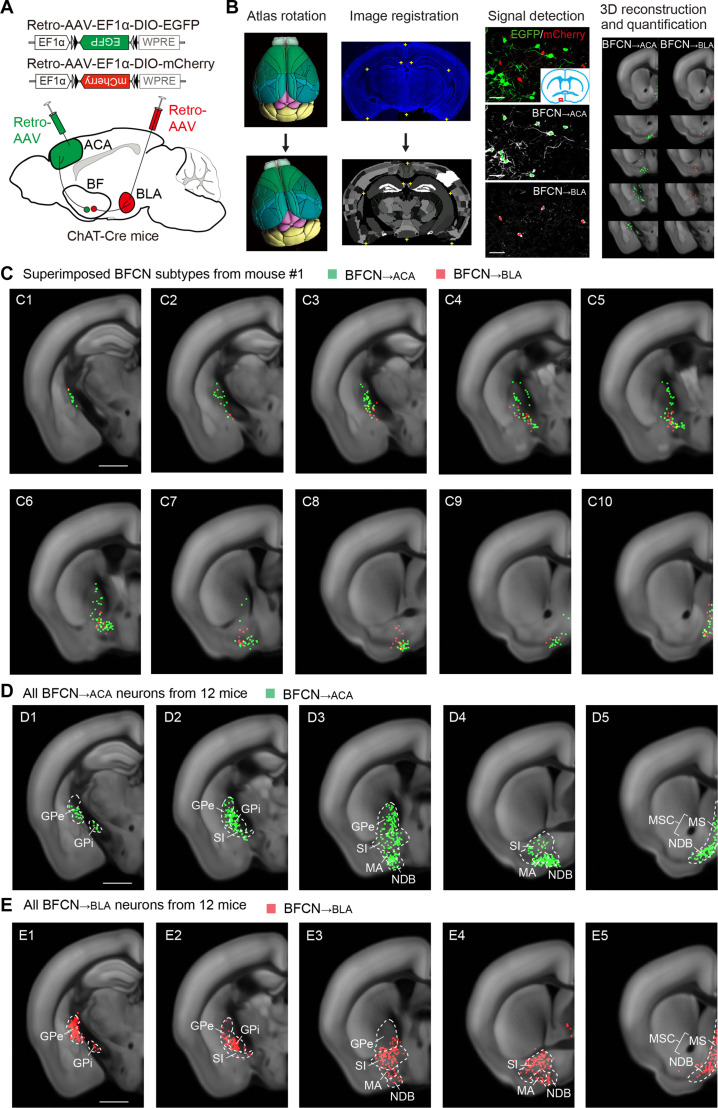
BFCN_→ACA_ and BFCN_→BLA_ neurons are two distinct subtypes of BFCN with unique firing patterns. (**A**) Viral vectors and injection procedure for Retro-AAV–mediated retrograde tracing, with two Retro-AAVs injected into the same mouse to label BFCNs projecting to the ACA and BLA. (**B**) Procedure for data analysis (See Materials and Methods). For atlas rotation, the Allen reference atlas was rotated to match the experimental brain’s section angle. For image registration, the original image (top) was mapped onto the corresponding coronal section of the rotated reference atlas (bottom). For signal detection, retrogradely labeled BFCN_→ACA_ (green) and BFCN_→BLA_ (red) neurons were manually marked (green and red crosses). The example image is from mouse #1. Scale bar, 60 μm. For 3D reconstruction and quantification, detected signals were mapped onto the Allen reference atlas and were quantified as the percentage of labeled neurons in each brain region. Example images showing the location of labeled BFCN_→ACA_ (green) and BFCN_→BLA_ (red) neurons from mouse #1. (**C**) Superimposed Retro-AAV–labeled BFCN_→ACA_ (green) and BFCN_→BLA_ (red) neurons from mouse #1, showing minimal overlap. Scale bar, 1 mm. (**D**) Summary of Retro-AAV–labeled BFCN_→ACA_ neurons in all samples (*n* = 12 mice). Green dots, detected BFCN_→ACA_ neurons. Scale bar, 1 mm. (**E**) Similar to (D), but for BFCN_→BLA_ neurons. Red dots, detected BFCN_→BLA_ neurons. Scale bar, 1 mm. Relevant abbreviations: GPe, external globus pallidus; GPi, internal globus pallidus; SI, substantia innominata; MA, magnocellular nucleus; NDB, diagonal band nucleus; MS, medial septal nucleus; MSC, medial septal complex.

Previous studies have suggested that the overlap rates of BFCNs targeting different cortical areas depend on the connectivity strength of their target areas (*5*, *24*). Given the sparse connection between the ACA and the BLA (*37*, *42*, *43*), we speculate that the BFCNs that innervate these two areas are distinct subtypes. To test this hypothesis, we used ChAT-Cre mice, in which cholinergic neurons expressing choline acetyltransferase (ChAT) also express Cre recombinase (*44*). We injected two retrograde Cre-inducible adeno-associated viruses (AAVs) into the same ChAT-Cre mouse: one expressing enhanced green fluorescent protein (EGFP) (Retro-AAV-EF1α-DIO-EGFP) into the ACA and the other expressing mCherry (Retro-AAV-EF1α-DIO-mCherry) into the BLA (Fig. 1A). Note that in this group of 12 mice, 6 were coinjected with an AAV expressing EGFP (AAV-hSyn-EGFP) along with the Retro-AAV-EF1α-DIO-EGFP into the ACA to verify the injection site in the ACA (fig. S1A). In addition, 9 of the 12 mice were coinjected with an AAV expressing mCherry (AAV-hSyn-mCherry) along with the Retro-AAV-EF1α-DIO-mCherry into the BLA to confirm the injection site in the BLA (fig. S1B).

After sectioning and imaging, we used an established processing pipeline (*35*, *37*) for digital brain image analysis (Fig. 1B). Initial identification confirmed the existence of two distinct subtypes of Retro-AAV–labeled BFCNs: BFCN_→ACA_ neurons, which express EGFP, and BFCN_→BLA_ neurons, which express mCherry (Fig. 1B). Upon reconstructing and superimposing the locations of BFCN_→ACA_ and BFCN_→BLA_ neurons, we noted extensive overlap in their spatial distributions throughout the ventral, medial, and dorsal PAL (PALv, PALm, and PALd), specifically (i) in the substantia innominata (SI) and magnocellular nucleus (MA) within the PALv; (ii) in the medial septal complex (MSC) within the PALm, which comprises the MS and the diagonal band nucleus (NDB); and (iii) in the external and internal globus pallidus segments (GPe and GPi) within the PALd (see Fig. 1, C and D; movie S1; and tables S1, S2, and S3). Note that the MS remains consistent between previous BF studies (*1*, *5*) and the Allen Mouse Brain Atlas. The vertical limb of the diagonal band and the horizontal limb of the diagonal band are combined into the NDB in the Allen Atlas. The nucleus basalis corresponds to the SI, MA, and part of the GPi in the Allen Atlas.

**Fig. 2.**
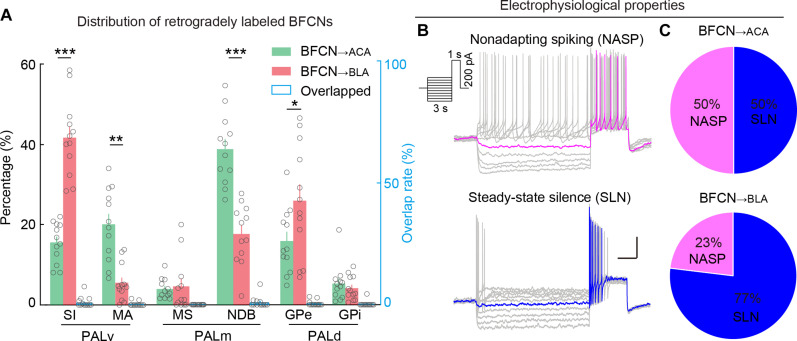
BFCN_→ACA_ and BFCN_→BLA_ neurons are two distinct subtypes of BFCN with unique firing patterns. (**A**) Distribution of retrogradely labeled BFCN_→ACA_ (green bar) and BFCN_→BLA_ (red bar) neurons (*n* = 12 mice). The distribution differs significantly across PAL subregions and BFCN subtypes [*F*_region_ (5,18) = 96, *P*_region_ = 3 × 10^−12^, *F*_region*type_ (5,18) = 15, *P*_region*type_ = 6 × 10^−6^, two-way mixed analysis of variance (ANOVA)]. There are significantly more BFCN_→BLA_ than BFCN_→ACA_ neurons in the SI and GPe (SI, *P* = 3 × 10^−8^; GPe, *P* = 0.04, Tukey’s post hoc test). In contrast, there are significantly more BFCN_→ACA_ than BFCN_→BLA_ neurons in the MA and NDB (MA, *P* = 0.001; NDB, *P* = 4 × 10^−5^). Notably, there was little overlap (blue bar) among the PAL subregions. Significant differences in the distribution between BFCN_→ACA_ and BFCN_→BLA_ neurons are indicated by asterisks. *P < 0.05; **P < 0.01; ***P < 0.001. Data are presented as the means ± SEM. (**B** and **C**) Intrinsic electrophysiologic properties of BFCN_→ACA_ and BFCN_→BLA_ neurons. BFCN_→ACA_, *n* = 40 neurons; BFCN_→BLA_, *n* = 26 neurons. (B) Top, firing pattern of an NASP neuron, showing NASP at the steady state on positive current injections. Bottom, firing pattern of a SLN neuron, showing a no-spiking period after the first few spikes. Scale bars, 500 ms, 20 mV. Inset, current steps used to elicit the firing patterns. (C) Pie chart illustrating the percentage of BFCN_→ACA_ and BFCN_→BLA_ neurons exhibiting NASP and SLN firing patterns.

Given that the number of labeled neurons varied across brains, and aiming to assess an equally weighted population average for each brain, we calculated the distribution of labeled BFCNs in each PAL subregion by dividing the number of labeled BFCNs in that region by the total number of labeled neurons in the PAL. We found that within each PAL subregion, there is little overlap between BFCN_→ACA_ and BFCN_→BLA_ neurons (overlap rates: SI, 1.3 ± 0.6%; MA, 0.4 ± 0.3%; MS, 0 ± 0%; NDB, 1.1 ± 0.7%; GPe, 0.6 ± 0.3%; GPi, 0.4 ± 0.4%), indicating that these are distinct subtypes (Fig. 2A and fig. S1).

BFCN_→ACA_ and BFCN_→BLA_ neurons exhibit distinct distribution patterns in PAL subregions. While both BFCN subtypes are similarly distributed in the MS and GPi, the SI and GPe contain significantly more BFCN_→BLA_ neurons than BFCN_→ACA_ neurons (in the SI, BFCN_→ACA_, 16 ± 1%; BFCN_→BLA_, 42 ± 3%; *P* = 3 × 10^−8^, Tukey’s post hoc test; in the GPe, BFCN_→ACA_, 16 ± 2%; BFCN_→BLA_, 26 ± 4%; *P* = 0.04; *n* = 12 mice; Fig. 2A). Conversely, the MA and NDB have significantly more BFCN_→ACA_ neurons than BFCN_→BLA_ neurons (in the MA, BFCN_→ACA_, 20 ± 4%; BFCN_→BLA_, 4 ± 2%; *P* = 0.001; in the NDB, BFCN_→ACA_, 39 ± 2%; BFCN_→BLA_, 18 ± 2%; *P* = 4 × 10^−5^; Fig. 2A). In addition, exchanging the Retro-AAV vectors injected into the ACA and BLA of ChAT-Cre mice—specifically, injecting Retro-AAV-EF1α-DIO-mCherry into the ACA and Retro-AAV-EF1α-DIO-EGFP into the BLA—revealed specific distribution patterns in PAL subregions that were similar to those observed before the exchange, along with low overlap rates between retrogradely labeled BFCN_→ACA_ and BFCN_→BLA_ neurons (figs. S2 and S3). Together, these results indicate that BFCN_→ACA_ and BFCN_→BLA_ neurons are distinct subtypes with unique distribution patterns in the PAL.

### BFCN_→ACA_ and BFCN_→BLA_ populations exhibit distinct firing patterns

BFCNs with various firing patterns have been shown to form functionally distinct circuits with sensory cortices and with the hippocampus that differentially modulate attention and fear (*45*, *46*). To explore the potential functional divergence between BFCN_→ACA_ and BFCN_→BLA_ neurons, we measured their firing patterns using whole-cell patch-clamp recordings on Retro-AAV–labeled BFCNs in acute brain slices from ChAT-Cre mice (BFCN_→ACA_, *n* = 40 neurons; BFCN_→BLA_, *n* = 26 neurons). Following a previous report (*45*), we applied an incremental 3-s “prepolarization” step current injection followed by a 1-s suprathreshold positive square pulse to assess neuronal firing from multiple internal states, starting from various hyperpolarized or depolarized membrane potentials.

In BFCN_→ACA_ and BFCN_→BLA_ neurons, we identified two distinct firing patterns: one displaying nonadapting spiking (NASP), and the other showing steady-state silence (SLN) with no-spiking period after the initial spikes (Fig. 2B and fig. S4). Compared to SLN-BFCNs, NASP-BFCNs exhibited significantly shorter no-spiking periods (spike silence) during the 1-s suprathreshold current injection, irrespective of the prepolarization current level (fig. S4B). Moreover, the prepolarization current level affected both spike delays and instantaneous firing rates of NASP- and SLN-BFCNs. Specifically, although both NASP- and SLN-BFCNs respond with minimal delays when depolarized, NASP-BFCNs showed significantly longer spike delays than SLN-BFCNs under hyperpolarized conditions. In depolarized conditions, NASP-BFCNs exhibited significantly lower instantaneous firing rates compared to SLN-BFCNs; such a difference was not observed under hyperpolarized conditions (fig. S4, C and D). These differences in firing patterns demonstrate functional distinctions between NASP- and SLN-BFCNs in hyperpolarized and depolarized internal states.

Both NASP- and SLN-BFCNs were detected in BFCN_→ACA_ and BFCN_→BLA_ neurons, and a chi-square test indicated that the distribution of NASP- and SLN-BFCNs within BFCN_→ACA_ and BFCN_→BLA_ neuron populations differs significantly (*P* = 0.03; Fig. 2C). BFCN_→ACA_ neurons comprise half NASP-BFCNs and half SLN-BFCNs (NASP, *n* = 20 neurons; SLN, *n* = 20 neurons), whereas BFCN_→BLA_ neurons comprise 30% NASP-BFCNs and 70% SLN-BFCNs (NASP, *n* = 6 neurons; SLN, *n* = 20 neurons). These results collectively establish that BFCN_→ACA_ and BFCN_→BLA_ neurons exhibit distinct firing patterns in response to excitatory inputs at the population level, supporting their potential function in transferring distinct information to downstream targets.

### BFCN_→ACA_ and BFCN_→BLA_ exhibit distinct whole-brain projection patterns

**Fig. 3.**
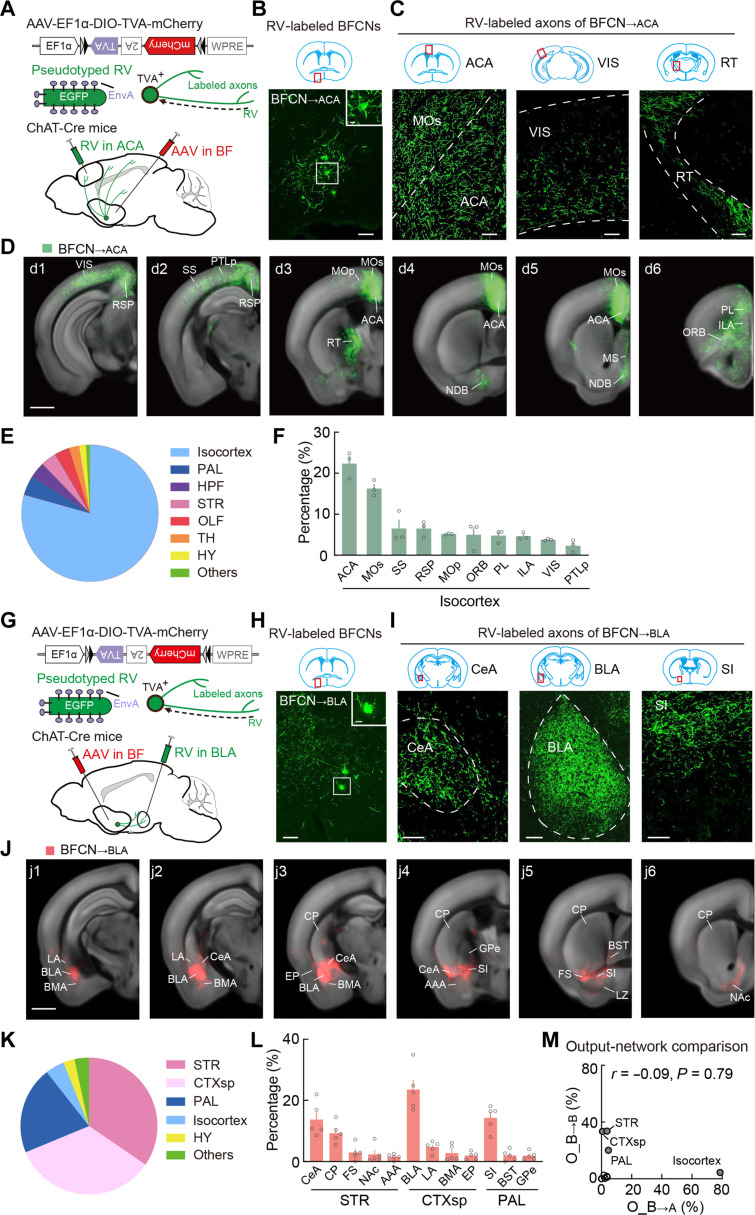
Whole-brain distributions of axonal projections from BFCN_→ACA_ and BFCN_→BLA_ neurons. (**A**) Viral vectors and injection procedure for RV-mediated tracing of axons from BFCN_→ACA_ neurons. (**B**) Fluorescence image of the BF (red box in coronal diagram) showing RV-labeled BFCN_→ACA_ neurons. Scale bar, 100 μm. Inset, enlarged view of the region in the white box. Scale bar, 20 μm. (**C**) Fluorescence images of RV-labeled axons of BFCN_→ACA_ neurons (green) in the ACA, VIS, and thalamic reticular nucleus (RT) (red box in coronal diagram). Immunostaining against EGFP was performed using tyramide signal amplification to enhance the fluorescence signal. Scale bars, 100 μm. (**D**) Axons of BFCN_→ACA_ neurons detected in all samples. Scale bar, 1 mm. (**E**) Pie chart illustrating the percentage of BFCN_→ACA_ axons distributed across the whole brain. (**F**) Percentages of labeled axons (See Materials and Methods) in the indicated isocortex subregions (*n* = 3 mice). Included are subregions with >1.5% labeling. Data are presented as the means ± SEM. (**G** to **L**) Similar to (A) to (F) but for BFCN_→BLA_ neurons. *n* = 5 mice. (**M**) Comparison of axon distributions of BFCN_→ACA_ and BFCN_→BLA_ neurons. Filled circles, strongly connected brain regions (i.e., with >10% labeling). Relevant abbreviations: PAL, pallidum; HPF, hippocampal formation; STR, striatum; OLF, olfactory areas; TH, thalamus; HY, hypothalamus; CTXsp, cortical subplate; ACA, anterior cingulate cortex; MOs, secondary motor cortex; SS, somatosensory cortex; RSP, retrosplenial cortex; MOp, primary motor cortex; ORB, orbital cortex; PL, prelimbic cortex; ILA, infralimbic cortex; VIS, visual cortex; PTLp, posterior parietal cortex; CeA, central amygdala; CP, caudoputamen; FS, fundus of striatum; NAc, nucleus accumbens; BLA, basolateral amygdala; LA, lateral amygdala; BMA, basomedial amygdala; EP, endopiriform nucleus; SI, substantia innominata; BST, bed nuclei of the stria terminalis; GPe, external globus pallidus.

Functional studies have shown that BFCNs coordinately modulate multiple downstream target areas to regulate cognition and emotion processing (*6*, *7*, *47*–*53*), and single-neuron anatomical studies have revealed that each BFCN innervates multiple downstream areas (*21*, *22*). To identify which brain areas are modulated by BFCN_→ACA_ and BFCN_→BLA_ neurons to execute their functions, we used RV-assisted axon mapping to delineate the output network of each subtype. For BFCN_→ACA_ neurons, we injected a Cre-inducible AAV expressing avian-specific retroviral receptor (TVA) (AAV-EF1α-DIO-TVA-mCherry) into the BF of ChAT-Cre mice, and a modified RV-expressing EGFP (CVS-N2cΔG-EGFP+EnvA) (*54*) into the ACA 2 weeks after AAV injection (Fig. 3A). This allowed RV to enter the TVA-expressing axon terminals of BFCN_→ACA_ neurons in the ACA, to be transported retrogradely to the BFCN_→ACA_ neurons, and to label all their axon collaterals with EGFP (Fig. 3, B and C). Since the modified RV lacks the rabies-virus glycoprotein (RVG) required for transsynaptic spread, no EGFP-expressing cells were observed outside the PAL (fig. S5). To enhance the visibility of labeled thin axons, we performed tyramide signal amplification for EGFP (Fig. 3C). “Long-range connections” are defined as those formed between BFCNs in the PAL and brain structures outside the PAL, in contrast to “local connections” within the PAL.

We found that BFCN_→ACA_ neurons extensively innervate the isocortex, with 79% of their total axons distributed in this region (Fig. 3, D and E; fig. S6; movie S2; and table S4). In addition to dense projections to the ACA, BFCN_→ACA_ neurons also target other cortical areas, including prefrontal cortices (orbital, prelimbic, and infralimbic cortex; ORB, PL, and ILA), medial association cortices (retrosplenial and posterior parietal cortex), motor cortices (primary and secondary motor cortex; MOs and MOp), and sensory cortices (somatosensory and visual cortex) (Fig. 3F). Notably, 22% of BFCN_→ACA_ axons were found in the ACA, 16% were in the MOs, and 5% were in the MOp (Fig. 3F). In addition, a dual Retro-AAV injection experiment identified a small population of BFCNs innervating both the ACA and MO (including MOp and MOs), with overlap rates ranging from 6 to 25% across PAL subregions, further supporting the presence of BFCNs projecting to both the ACA and MO (fig. S7).We know from previous studies that cortical areas are hierarchically organized; higher-level areas such as prefrontal cortices integrate information from lower-level sensory and motor areas and execute behavior-related top-down modulations, including attention modulation (*35*, *55*). Our observation of extensive innervation of cortical areas by BFCN_→ACA_ neurons suggests their role in coordinately modulating cortical functions across hierarchical levels, potentially in the top-down attention modulation of sensorimotor processing.

For BFCN_→BLA_ neurons, we injected Cre-inducible AAV expressing TVA (AAV-EF1α-DIO-TVA-mCherry) into the BF of ChAT-Cre mice, followed by an injection of modified RV (CVS-N2cΔG-EGFP+EnvA) into the BLA 2 weeks after AAV injection (Fig. 3, G to I). BFCN_→BLA_ neurons primarily innervate the STR, CTXsp, and PAL, with 34, 33, and 20% of total axons distributed in these regions, respectively (Fig. 3, J and K; movie S2; and table S4). BFCN_→BLA_ neurons send long-range projections to the caudoputamen (CP) of the STR and send local projections to the SI of the PAL (Fig. 3L). Moreover, BFCN_→BLA_ neurons extensively project to subregions of the amygdala, with, for example, projections to the BLA of the CTXsp (24%) and to the central amygdalar nucleus (CeA) of the STR (14%) (Fig. 3L). The extensive innervations of amygdala subregions by BFCN_→BLA_ neurons suggest that these neurons modulate emotion processing–related amygdalar functions.

We also compared the output networks of BFCN_→ACA_ and BFCN_→BLA_ neurons, which are defined as the regions throughout the brain receiving the outputs of these BFCNs. We calculated the Pearson correlation coefficient (CC) across brain samples for the averaged spatial distribution values, using the 11 major regions defined in the Allen Mouse Brain Atlas (*56*), including the isocortex, olfactory areas, hippocampal formation (HPF), CTXsp, STR, PAL, TH, HY, MB, hindbrain (HB), and cerebellum (CB). High CCs among individual samples in each group—BFCN_→ACA_ (0.997 ± 0.001, *P* < 2 × 10^−10^, *n* = 3 mice) and BFCN_→BLA_ (0.92 ± 0.02, *P* < 0.005, *n* = 5 mice)—support the reproducibility of our output mapping and highlight consistent projection patterns for each BFCN subtype. In contrast, a near-zero CC (CC = −0.09) between the BFCN_→ACA_ and BFCN_→BLA_ neurons reflects their distinct projection patterns throughout the brain (Fig. 3M). That is, BFCN_→ACA_ neurons target the isocortex, whereas BFCN_→BLA_ neurons target the STR, CTXsp, and PAL. This divergence in projection targets between the two BFCN subtypes indicates their distinct whole-brain output networks that apparently operate independently in cholinergic modulation of cognition and emotion.

As noted in previous studies, when Cre-inducible AAV expressing TVA and modified RV are coinjected at the same site, the very low-level of leaky TVA expression in non–Cre-expressing cells can lead to RV infection of these cells (owing to the highly efficient interaction between TVA and modified RV) (*57*, *58*). To assess whether such contamination occurred under our experimental conditions, we performed control experiments by injecting AAV-EF1α-DIO-TVA-mCherry into the BF of wild-type mice, followed 2 weeks later by injecting modified RV (CVS-N2cΔG-EGFP+EnvA) into the ACA or BLA. No EGFP expression was observed in the BF, indicating that leaky TVA expression did not interfere with the detection of BFCN projections under our conditions (fig. S8).

### Cortical, MB, and hypothalamic excitatory inputs converge with subcortical inhibitory inputs in BFCN_→ACA_

**Fig. 4.**
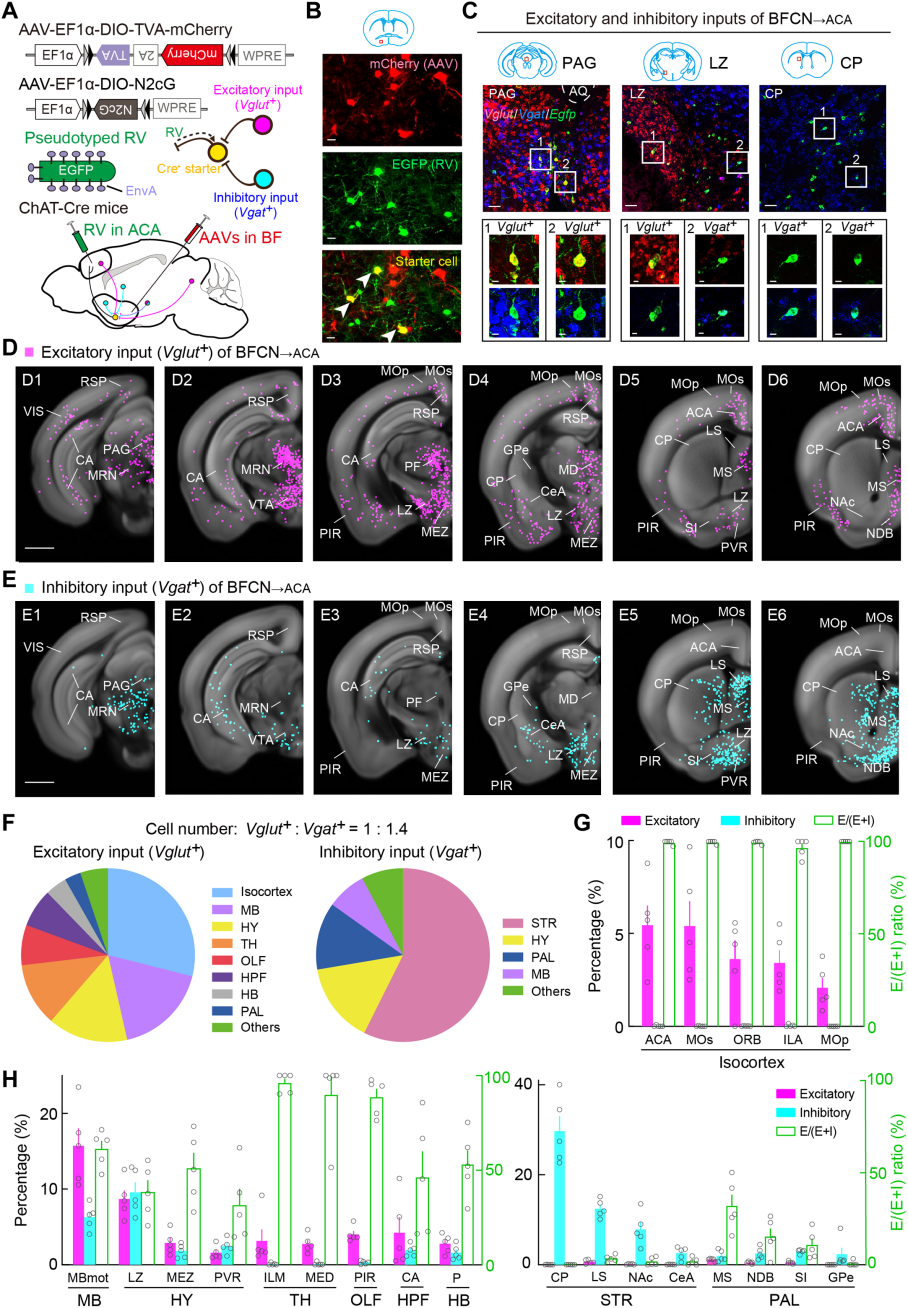
Whole-brain distributions of excitatory and inhibitory inputs to BFCN_→ACA_ neurons. (**A**) Viral vectors and injection procedure for RV-mediated transsynaptic retrograde tracing from BFCN_→ACA_ neurons. (**B**) Fluorescence images of the BF (red box in coronal diagram), showing starter cells (yellow). Scale bar, 20 μm. Green, EGFP; red, mCherry. (**C**) Retrogradely labeled input neurons (green) in the periaqueductal gray (PAG), LZ, and CP with RNAscope in situ hybridization of *Vglut* (red) and *Vgat* (blue). Top, fluorescent images of the PAG, LZ, and CP. Scale bars, 50 μm. Bottom, enlarged view of regions in white boxes, showing RV-labeled neurons expressing *Vglut* or *Vgat*. Scale bars, 10 μm. (**D**) Excitatory input neurons (*Egfp^+^* and *Vglut^+^*) of BFCN_→ACA_ neurons detected in all samples (*n* = 5 mice). Scale bar, 1 mm. (**E**) Similar to (D) but for inhibitory input neurons (*Egfp^+^* and *Vgat^+^*). (**F**) Pie charts illustrating the percentage of excitatory (left) and inhibitory (right) inputs to BFCN_→ACA_ neurons distributed across the whole brain. (**G**) Percentages of excitatory (magenta bars) and inhibitory (cyan bars) inputs in the indicated isocortex subregions (See Materials and Methods). Included are subregions with >2% labeling. Green bars indicate the E/(E + I) ratio. Data are presented as the means ± SEM. (**H**) Similar to (G) but for subregions of other major brain regions with >2% labeling. Relevant abbreviations: MB, midbrain; HY, hypothalamus; TH, thalamus; OLF, olfactory areas; HPF, hippocampal formation; HB, hindbrain; PAL, pallidum; STR, striatum; ACA, anterior cingulate cortex; MOs, secondary motor cortex; ORB, orbital cortex; ILA, infralimbic cortex; MOp, primary motor cortex; MBmot, motor-related MB; LZ, lateral hypothalamic zone; MEZ, medial hypothalamic zone; PVR, paraventricular nucleus; ILM, intralaminar thalamic nuclei; MED, medial group of dorsal thalamus; PIR, piriform area; CA, Ammon’s horn; P, Pons; CP, caudoputamen; LS, lateral septal nucleus; NAc, nucleus accumbens; CeA, central amygdala; MS, medial septal nucleus.

Previous studies have mapped whole-brain inputs to BFCNs (*24*–*26*), although the specific identities of these input neurons (i.e., whether excitatory or inhibitory) remain ambiguous. We combined RV-assisted trans-monosynaptic retrograde tracing (*54*, *59*) and triple-plex RNAscope in situ hybridization to pinpoint the identities of input neurons. To map the monosynaptic inputs to BFCN_→ACA_ neurons, we injected Cre-inducible AAVs expressing TVA, mCherry, and RVG (AAV-EF1α-DIO-TVA-mCherry and AAV-EF1α-DIO-N2cG) into the BF of ChAT-Cre mice. Two weeks later, a modified RV (CVS-N2cΔG-EGFP+EnvA) was injected into the ACA. This viral strategy allowed RV to enter the TVA-expressing axon terminals of BFCN_→ACA_ neurons in the ACA, to be transported retrogradely to the BFCN_→ACA_ neurons and to trans-monosynaptically and retrogradely label the input neurons to BFCN_→ACA_ neurons with the help of RVG (Fig. 4, A and B, and figs. S9 and S10). Subsequent triple-plex RNAscope in situ hybridization targeting *EGFP* and selected gene markers for excitatory (*vesicular glutamate transporter 1* and *2*, *Vglut1* and *Vglut2*) and inhibitory (*vesicular GABA transporter*, *Vgat*) neurons were used to ascertain the identity of input neurons (Fig. 4C).

We generated three-dimensional (3D) visualizations and performed quantitative analyses of the distributions of excitatory (*Vglut^+^*) and inhibitory (*Vgat^+^*) input neurons targeting BFCN_→ACA_ neurons throughout the brain (Fig. 4, D to F; movie S3; and table S5). We found high CCs for both input types across brain samples for the averaged spatial distribution values for the 11 major regions defined in the Allen Mouse Brain Atlas (excitatory inputs, 0.85 ± 0.02, *P* < 0.01; inhibitory inputs, 0.98 ± 0.01; *P* < 2 × 10^−5^; *n* = 5 mice), indicating reproducibility and consistent input patterns. Across the whole brain, there are more inhibitory input neurons than excitatory input neurons (*Vglut^+^* excitatory neurons: 2166 ± 605; *Vgat^+^* inhibitory neurons: 3091 ± 645; *P* = 0.08, paired *t* test). We also calculated the *E*/(*E* + I) ratio for each brain region to assess the distribution of excitatory and inhibitory inputs provided by those regions. We noted that excitatory and inhibitory input neurons targeting BFCN_→ACA_ neurons originate from distinct brain regions: The isocortex and MB provide excitatory inputs (29 and 17%, respectively), while the STR and PAL provide inhibitory inputs (57 and 13%, respectively) (Fig. 4F). Notably, the HY provides both excitatory and inhibitory inputs, contributing ~15% of each type (Fig. 4F).

We subsequently cataloged all specific subregions having >2% of total excitatory or inhibitory inputs to BFCN_→ACA_ neurons (Fig. 4, G and H), using similar criteria as a previous study (*37*). Among excitatory-input sources, the subregions of the isocortex (e.g., ACA, MOs, ORB, ILA, and MOp) exclusively provide excitatory inputs (5, 5, 4, 3, and 2%) (Fig. 4G). The motor-related MB (MBmot) of the MB provides 16% of excitatory inputs (Fig. 4H). Among inhibitory input sources, subregions of the STR, including the CP, lateral septal nucleus (LS), nucleus accumbens (NAc), and CeA, exclusively provide inhibitory inputs (30, 12, 8, and 3%) (Fig. 4H). Similarly, the subregions of the PAL, including the MS, NDB, SI, and GPe provide inhibitory inputs (2, 3, 3, and 2%) (Fig. 4H). Within the HY, which provides both types of inputs, the lateral hypothalamic zone (LZ) provides 9% excitatory and 10% inhibitory inputs; the medial hypothalamic zone (MEZ) provides 3% excitatory inputs and 2% inhibitory inputs; and the paraventricular nucleus (PVR) provides 2% of inhibitory inputs (Fig. 4H).

Our finding that inhibitory inputs of BFCN_→ACA_ neurons are from subcortical areas (e.g., STR, HY, and PAL) aligns with the major input sources identified in the aforementioned mapping studies. Expanding beyond this, by distinguishing between input neuron types, our findings delineate the unique excitatory- and inhibitory-input networks for BFCN_→ACA_ neurons, revealing, for example, that cortical, MB, and hypothalamic excitatory inputs converge with subcortical inhibitory inputs in these neurons. Notably, while the MB and HY contain both excitatory and inhibitory neurons, our results establish that these regions together provide more excitatory input neurons to BFCN_→ACA_ neurons than the isocortex, suggesting previously unappreciated roles of these regions in activating BFCN_→ACA_ neurons.

### BFCN_→ACA_ output network selectively interacts with its excitatory-input network

**Fig. 5.**
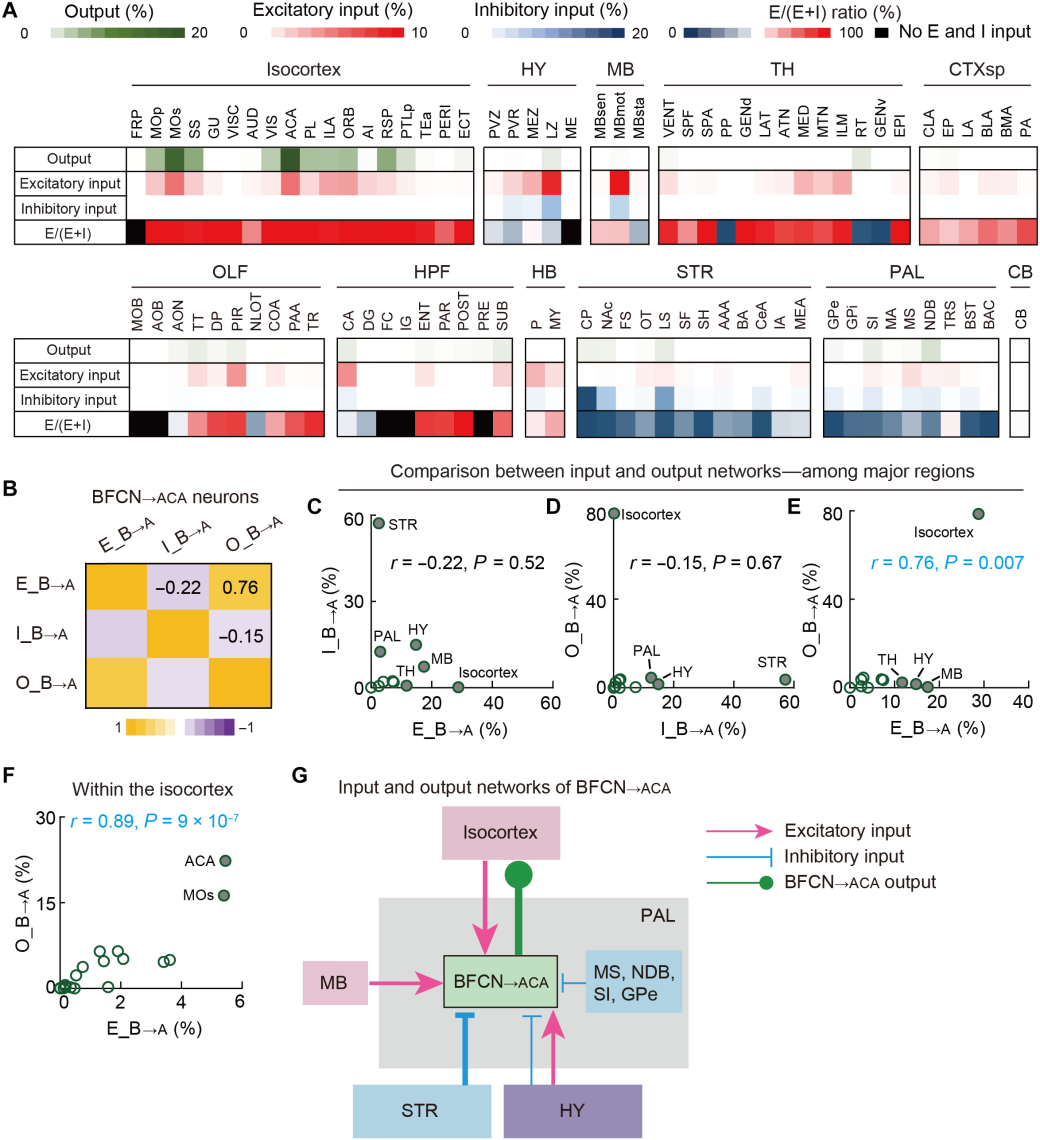
The BFCN_→ACA_ output network selectively interacts with the BFCN_→ACA_ excitatory-input network. (**A**) Matrixes showing the percentages of outputs (axons), excitatory inputs, inhibitory inputs, and the E/(E + I) ratio of brain subregions for BFCN_→ACA_ neurons. Rows represent data averaged from the experiments examining BFCN_→ACA_ neurons, while columns list subregions with either >1.5% output labeling or >2% input labeling (excitatory or inhibitory). (**B**) Matrix of CCs between input and output networks of BFCN_→ACA_ neurons. (**C**) The percentage of inhibitory inputs versus the percentage of excitatory inputs in major brain regions for BFCN_→ACA_ neurons. Filled circles indicate strongly connected regions (i.e., with >10% labeling of excitatory or inhibitory inputs). (**D**) The percentage of outputs versus the percentage of inhibitory inputs in major brain regions for BFCN_→ACA_ neurons. Filled circles indicate strongly connected regions (i.e., with >10% labeling of outputs or inhibitory inputs). (**E**) The percentage of outputs versus the percentage of excitatory inputs in major brain regions for BFCN_→ACA_ neurons. Filled circles indicate strongly connected regions (i.e., with >10% labeling of outputs or excitatory inputs). (**F**) The percentage of outputs versus the percentage of excitatory inputs in the indicated subregions of the isocortex. Filled circles indicate strongly connected subregions (i.e., with >8% labeling of outputs or >4% labeling of excitatory inputs). (**G**) Diagrams illustrating the input and output networks of BFCN_→ACA_ neurons.

Previous studies have shown that input neurons to BFCNs projecting to various cortical areas exhibit specific interaction patterns, such as strong somatosensory cortical inputs to motor cortex–projecting BFCNs (*1*, *5*, *24*). To investigate the interactions among three types of BFCN_→ACA_ neuron networks (excitatory input, inhibitory input, and output), we calculated the CC across brain samples using matrices representing the distribution of inputs and outputs in 11 major regions (the same as in Fig. 3M) defined in the Allen Mouse Brain Atlas (Fig. 5, A and B). The low CC between the excitatory- and inhibitory-input networks (CC = −0.22) and the low CC between the inhibitory-input and output networks (CC = −0.15) (Fig. 5, C and D) indicate minimal interactions, suggesting that these networks likely operate independently.

In contrast, a high positive CC (CC = 0.76) between the excitatory-input and output networks indicates reciprocal connections between BFCN_→ACA_ neurons and their excitatory-input sources, particularly with the isocortex (Fig. 5E). Upon calculating CCs for interactions within isocortex subregions, we found an even higher CC of 0.89, indicating that the cortical areas receiving relatively more extensive cholinergic projections from BFCN_→ACA_ neurons also reciprocally provide more excitatory inputs back to them (Fig. 5F). For example, the ACA and MOs, which receive the largest amount of outputs from BFCN_→ACA_ neurons, correspondingly provide the most excitatory inputs back to these neurons (Fig. 5F). Our results collectively indicate unique interaction patterns between the input and output networks of BFCN_→ACA_ neurons (diagram shown in Fig. 5G): the output network of BFCN_→ACA_ neurons preferentially interacts with the BFCN_→ACA_ excitatory-input network, characterized by extensive reciprocal connections between these neurons and the isocortex. This is a plausible arrangement through which BFCN_→ACA_ neurons could modulate their excitatory cortical inputs.

### Cortical, thalamic, and amygdalar excitatory inputs converge with subcortical inhibitory inputs in BFCN_→BLA_

**Fig. 6.**
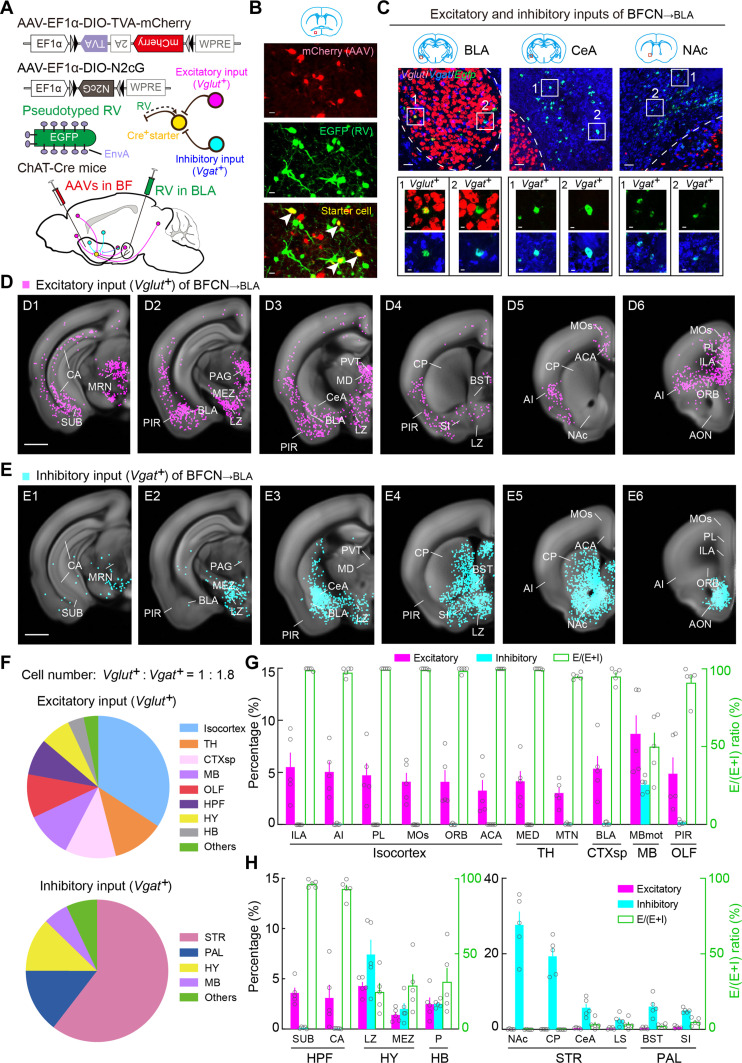
Whole-brain distributions of excitatory and inhibitory inputs to BFCN_→BLA_ neurons. (**A**) Viral vectors and injection procedure for RV-mediated transsynaptic retrograde tracing from BFCN_→BLA_ neurons. (**B**) Fluorescence images of the BF (red box in coronal diagram), showing starter cells (yellow). Scale bar, 20 μm. Green, EGFP; red, mCherry. (**C**) Retrogradely labeled input neurons (green) in the BLA, CeA, and NAc with RNAscope in situ hybridization of *Vglut* (red) and *Vgat* (blue). Top, fluorescent images of the BLA, CeA, and NAc. Scale bars, 50 μm. Bottom, enlarged view of regions in white boxes, showing RV-labeled neurons expressing *Vglut* or *Vgat*. Scale bars, 10 μm. (**D**) Excitatory input neurons (*Egfp^+^* and *Vglut^+^*) of BFCN_→BLA_ neurons detected in all samples (*n* = 5 mice). Scale bar, 1 mm. (**E**) Similar to (D) but for inhibitory input neurons (*Egfp^+^* and *Vgat^+^*). (**F**) Pie charts illustrating the percentage of excitatory (top) and inhibitory (bottom) inputs to BFCN_→BLA_ neurons distributed across the whole brain. (**G**) Percentages of excitatory (magenta bars) and inhibitory (cyan bars) input in the indicated subregions of the isocortex, TH, CTXsp, MB, and OLF. Included are subregions with >2% labeling. Green bars indicate the E/(E + I) ratio. Data are presented as the means ± SEM. (**H**) Similar to (G) but for subregions of other major brain regions with >2% labeling. Relevant abbreviations for subregions: ILA, infralimbic cortex; AI, agranular insular cortex; PL, prelimbic cortex; MOs, secondary motor cortex; ORB, orbital cortex; ACA, anterior cingulate cortex; MED, medial group of dorsal thalamus; MTN, midline group of dorsal thalamus; BLA, basolateral amygdala; MBmot, motor-related MB; PIR, piriform area; SUB, subiculum; CA, Ammon’s horn; LZ, lateral hypothalamic zone; MEZ, medial hypothalamic zone; P, Pons; NAc, nucleus accumbens; CP, caudoputamen; CeA, central amygdala; LS, lateral septal nucleus; BST, bed nuclei of the stria terminalis; SI, substantia innominate.

A previous study (without input neuron type differentiation) reported that the STR is the largest input source to BFCN_→BLA_ neurons and noted that these BFCNs receive more amygdalar inputs than BFCNs targeting the isocortex (*24*). To investigate the distribution of excitatory and inhibitory inputs of BFCN_→BLA_ neurons, we applied similar viral strategies as those used for mapping BFCN_→ACA_ inputs but with RV injected into the BLA (Fig. 6, A and B, and fig. S11). Akin to BFCN_→ACA_ inputs, BFCN_→BLA_ neurons exhibit more inhibitory than excitatory input neurons throughout the brain (*Vglut^+^* excitatory neurons: 2571 ± 850; *Vgat^+^* inhibitory neurons: 4604 ± 1300; *P* = 0.03, paired *t* test), supporting the previously suggested notion that BFCNs are under strong inhibitory control (*1*, *5*).

We observed consistent patterns of excitatory and inhibitory inputs for BFCN_→BLA_ neurons, as evidenced by high CCs across brain samples (excitatory inputs, 0.84 ± 0.02, *P* < 0.01; inhibitory inputs, 0.97 ± 0.01, *P* < 3 × 10^−5^; *n* = 5 mice; Fig. 6, C to E, and movie S4). Similar to BFCN_→ACA_ neurons, BFCN_→BLA_ neurons receive extensive excitatory inputs from the isocortex (34%; Fig. 6F and table S6). The subregions of the isocortex—e.g., the ILA, PL, MOs, ORB, ACA, and agranular insular area (AI)—provide only excitatory inputs (6, 5, 4, 4, 3, and 5%) (Fig. 6G). In contrast to the excitatory inputs from the MB and HY to BFCN_→ACA_ neurons, we found that BFCN_→BLA_ neurons receive excitatory inputs from the TH (12%) and CTXsp (11%), especially from the medial group and midline group of the dorsal TH (MED and MTN, 4 and 3%) and from the BLA of the CTXsp (5%) (Fig. 6G).

The inhibitory input sources for BFCN_→BLA_ neurons are the same as those of BFCN_→ACA_ neurons: BFCN_→BLA_ neurons also receive inhibitory inputs from the STR, PAL, and HY (60, 14, and 12%). The HY provides both excitatory and inhibitory inputs with a bias toward inhibitory input neurons [E/(E + I) ratio = 24%], while the STR and PAL exclusively provide inhibitory inputs (Fig. 6H). Within the HY, the LZ provides 4% of excitatory inputs and 7% of inhibitory inputs, and the MEZ provide 2% of inhibitory inputs. Within the STR, the NAc and CP, respectively, provide 28 and 19% of inhibitory inputs, followed by the CeA and LS (6 and 2%). In addition, BFCN_→BLA_ neurons also receive inhibitory inputs from the bed nuclei of the stria terminalis (BST) and SI of the PAL (6 and 5%).

Our findings indicate that the inhibitory inputs to BFCN_→BLA_ neurons originate from subcortical areas similar to those of BFCN_→ACA_ neurons (e.g., the STR, HY, and PAL), consistent with major input sources identified in previous mapping studies (*24*–*26*). Moreover, the amygdalar subregions (e.g., the BLA and CeA) specifically provide inputs to BFCN_→BLA_ neurons but provide few inputs to BFCN_→ACA_ neurons, reinforcing the previous finding that BFCN_→BLA_ neurons receive more amygdalar inputs than BFCNs projecting to the isocortex. In addition, our data establish that the BLA provides excitatory inputs, whereas the CeA provides inhibitory inputs, suggesting distinct roles in modulating BFCN_→BLA_ neuron activity. Our data also delineate the unique excitatory- and inhibitory-input networks for BFCN_→BLA_ neurons, revealing that cortical, thalamic, and amygdalar excitatory inputs converge with subcortical inhibitory inputs in these neurons. The relatively high abundance of excitatory inputs from the isocortex to BFCN_→BLA_ neurons suggests top-down cortical control in cholinergic modulation of emotion processing.

### BFCN_→BLA_ output network selectively interacts with its inhibitory-input network

**Fig. 7.**
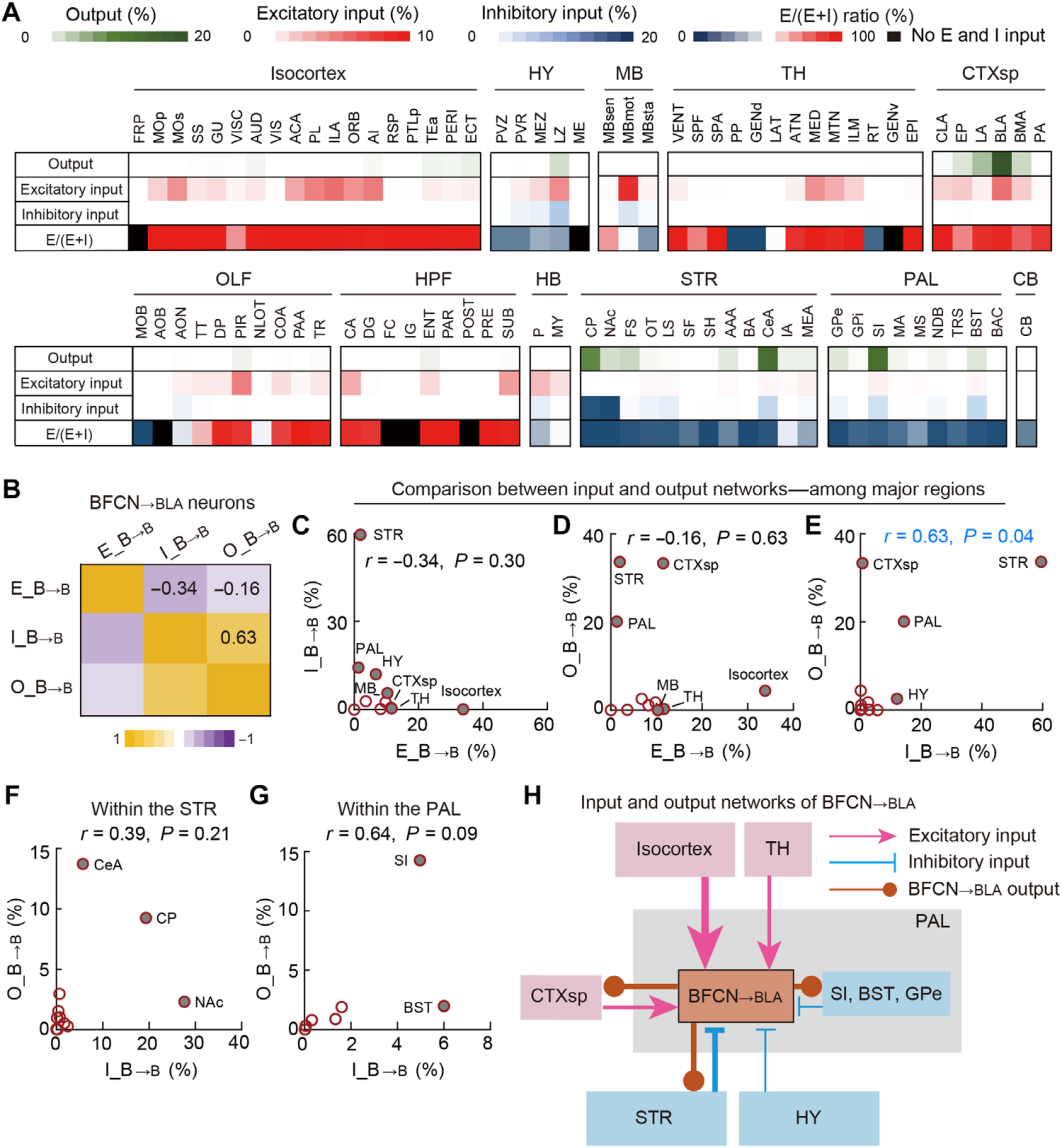
The BFCN_→BLA_ output network selectively interacts with the BFCN_→BLA_ inhibitory-input network. (**A**) Matrixes showing the percentages of outputs (axons), excitatory inputs, inhibitory inputs, and the E/(E + I) ratio of brain subregions for BFCN_→BLA_ neurons. Rows represent data averaged from the experiments examining BFCN_→BLA_ neurons, while columns list subregions with either >1.5% output labeling or >2% input labeling (excitatory or inhibitory). (**B**) Matrix of CCs between input and output networks of BFCN_→BLA_ neurons. (**C**) The percentage of inhibitory inputs versus the percentage of excitatory inputs in major brain regions for BFCN_→BLA_ neurons. Filled circles indicate strongly connected regions (i.e., with >10% labeling of excitatory or inhibitory inputs). (**D**) The percentage of outputs versus the percentage of excitatory inputs in major brain regions for BFCN_→BLA_ neurons. Filled circles indicate strongly connected regions (i.e., with >10% labeling of outputs or excitatory inputs). (**E**) The percentage of outputs versus the percentage of inhibitory inputs in major brain regions for BFCN_→BLA_ neurons. Filled circles indicate strongly connected regions (i.e., with >10% labeling of outputs or inhibitory inputs). (**F**) The percentage of outputs versus the percentage of inhibitory inputs in subregions of the STR. Filled circles indicate strongly connected subregions (i.e., with >4% labeling of outputs or >4% labeling of inhibitory inputs). (**G**) Similar to (F) but for subregions of the PAL. Filled circles indicate strongly connected subregions (i.e., with >4% labeling of outputs or >4% labeling of inhibitory inputs). (**H**) Diagrams illustrating the input and output networks of BFCN_→BLA_ neurons.

To investigate the interactions among the excitatory-input, inhibitory-input, and output networks of BFCN_→BLA_ neurons, we used a similar analytical framework as used for BFCN_→ACA_ neurons. Upon calculating CCs of averaged spatial distributions across these networks, our analysis revealed minimal interactions between the excitatory- and inhibitory-input networks, as indicated by a low CC of −0.34 (Fig. 7, A to C). Notably, a similarly low CC (CC = −0.16) between the excitatory-input and output networks indicates a lack of interactions among these networks, contrasting sharply with the extensive reciprocal interactions observed for BFCN_→ACA_ neurons (Fig. 7D).

In contrast to the minimal interactions between the excitatory-input and output networks for BFCN_→BLA_ neurons, the high positive CC (0.63) we found between their inhibitory-input and output networks indicates extensive reciprocal connections, particularly with the STR and PAL (Fig. 7E). A subregion analysis of the STR and PAL revealed diverse positive CCs of 0.39 and 0.64, indicating that while reciprocal connections exist, the extent of cholinergic outputs received and inhibitory inputs provided varies across these subregions, likely reflecting region-specific modulation of inhibitory inputs by BFCN_→BLA_ neurons (Fig. 7, F and G). These results delineate distinct interaction patterns between the input and output networks of BFCN_→BLA_ neurons (diagram shown in Fig. 7H): Unlike the selective interaction between the BFCN_→ACA_ output network and the BFCN_→ACA_ excitatory network, the output network of BFCN_→BLA_ neurons preferentially interacts with the BFCN_→BLA_ inhibitory-input network, which should in theory enable BFCN_→BLA_ neurons to effectively modulate their inhibitory inputs.

### Subcortical regions differ in excitatory/inhibitory input to both BFCN subtypes

Previous studies have reported putative excitatory inputs from the isocortex and inhibitory inputs from the STR to BFCNs based on the predominant neuron types in these regions (>80% of neurons are either *Vglut^+^* or *Vgat^+^*) (*24*–*26*). However, the organization of excitatory versus inhibitory inputs to BFCNs in their input regions has not been characterized. To assess whether brain regions differentially contribute excitatory versus inhibitory inputs to BFCN_→ACA_ and BFCN_→BLA_ neurons, we compared the distribution of these inputs across major brain regions. Note that our analysis initially focused on 9 of 11 examined major brain regions, specifically those with E + I >5% labeling for both BFCN_→ACA_ and BFCN_→BLA_ neurons. We excluded the CB and CTXsp in this analysis as our data indicated that the CB contributes minimal inputs to either BFCN subtype (BFCN_→ACA_ neurons: 0.01% of their excitatory inputs and 0.06% of inhibitory inputs; BFCN_→BLA_ neurons: 0.02% of their excitatory inputs and 0.02% of inhibitory inputs). The CTXsp provides many more excitatory inputs to BFCN_→BLA_ as compared to BFCN_→ACA_ neurons (BFCN_→ACA_ neurons: 2.6% of their excitatory inputs and 0.6% of inhibitory inputs; BFCN_→BLA_ neurons: 11.5% of their excitatory inputs and 0.7% of inhibitory inputs).

Chi-square tests indicated that the HY, MB, PAL, and HB contain significantly larger proportions of excitatory input neurons to BFCN_→ACA_ neurons than to BFCN_→BLA_ neurons (HY, *P* = 0.015; MB, *P* = 0.047; PAL, *P* = 0.008; HB, *P* = 0.002; fig. S12A), suggesting that inputs from these regions to BFCN_→ACA_ neurons are biased toward excitation relative to their inputs to BFCN_→BLA_ neurons. In contrast, the HPF contains a significantly larger proportion of inhibitory input neurons to BFCN_→ACA_ neurons than to BFCN_→BLA_ neurons (HPF, *P* = 5 × 10^−8^), indicating that HPF inputs to BFCN_→ACA_ neurons are biased toward inhibition relative to its inputs to BFCN_→BLA_ neurons.

We subsequently examined subregions of the aforementioned regions that are biased toward excitation for BFCN_→ACA_ neurons. Chi-square tests indicated that the LZ, MEZ, and PVR of the HY; the ventral tegmental area (VTA) of the MBmot; the NDB of the PAL; and the pons (P) and medulla of the HB contain significantly larger proportions of excitatory input neurons to BFCN_→ACA_ neurons than to BFCN_→BLA_ neurons (*P* < 0.048; fig. S12B). Given the established roles of these structures in modulating brain state and emotion processing (*1*, *5*, *60*–*67*), their preferential excitation of BFCN_→ACA_ neurons may facilitate brain-state– and emotion-related cholinergic modulation of cognition.

We also performed a subregion analysis of the HPF. The Ammon’s horn (CA), subiculum, and entorhinal area of the HPF contain significantly larger proportions of inhibitory input neurons to BFCN_→ACA_ neurons than to BFCN_→BLA_ neurons (*P* < 6 × 10^−3^, chi-square test; fig. S12C). Notably, while the CA consists of ~80% excitatory neurons and ~20% inhibitory neurons (*28*–*30*), CA inputs to BFCN_→ACA_ neurons are approximately half excitatory and half inhibitory, suggesting a specific role for CA inhibitory neurons in suppressing BFCN_→ACA_ neurons (fig. S12C). Together, these results highlight the apparently region-specific organization of excitatory versus inhibitory inputs in subcortical regions innervating both BFCN subtypes.

### A long-range reciprocal input-output loop connects BFCN_→ACA_ and BFCN_→BLA_ networks

Previous studies have demonstrated that BFCNs orchestrate the activities of multiple downstream target areas to regulate cognition and emotion processing (*7*, *47*–*53*). Given our findings that BFCN_→ACA_ and BFCN_→BLA_ neurons project to distinct downstream areas related to cognitive and emotional functions across the brain, we investigated the interactions among their input and output networks, which potentially contribute to the coordinated cholinergic modulation of cognition and emotion. The low CC between the BFCN_→ACA_ excitatory-input and BFCN_→BLA_ inhibitory-input networks (CC = −0.25) and the low CC between the BFCN_→ACA_ inhibitory-input and BFCN_→BLA_ excitatory-input networks (CC = −0.34) collectively indicate minimal interactions among these networks (figs. S13 and S14). These results suggest that the excitatory-input network of BFCN_→ACA_ neurons operates independently from the inhibitory-input network of BFCN_→BLA_ neurons, and vice versa.

**Fig. 8.**
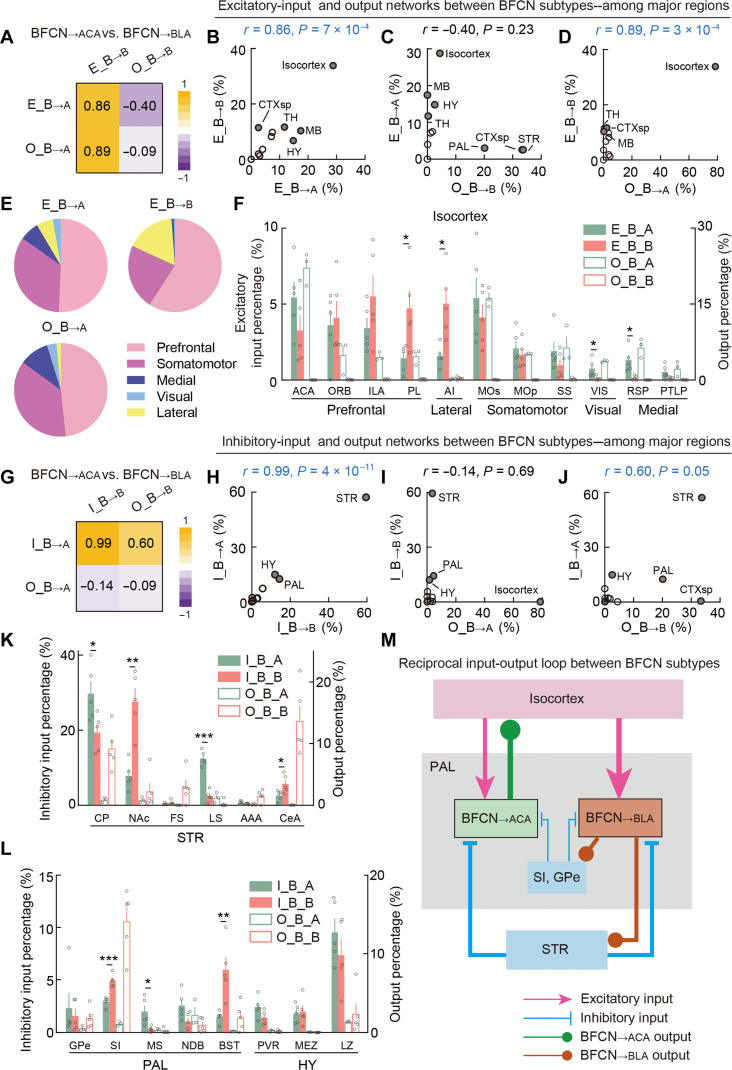
Long-range reciprocal input-output loop between BFCN_→ACA_ and BFCN_→BLA_ networks. (**A**) Matrix of CCs among excitatory-input and output networks between BFCN_→ACA_ and BFCN_→BLA_ neurons. (**B**) Percentages of excitatory inputs of BFCN_→BLA_ neurons versus percentages of excitatory inputs of BFCN_→ACA_ neurons in major brain regions. Filled circles indicate strongly connected regions (i.e., with >10% labeling of excitatory inputs for either BFCN_→BLA_ or BFCN_→ACA_ neurons). (**C**) Percentages of excitatory inputs of BFCN_→ACA_ neurons versus percentages of outputs of BFCN_→BLA_ neurons. Filled circles indicate strongly connected regions (i.e., with >10% labeling of excitatory inputs or >10% labeling of outputs). (**D**) Similar to (C) but for percentages of excitatory inputs of BFCN_→BLA_ neurons versus percentages of outputs of BFCN_→ACA_ neurons. (**E**) Pie charts illustrating percentages of excitatory inputs of BFCN_→ACA_ and BFCN_→BLA_ neurons and outputs of BFCN_→ACA_ neurons distributed in different cortical areas. (**F**) Percentages of excitatory inputs (solid bars) and outputs (empty bars) for BFCN_→ACA_ (green) and BFCN_→BLA_ (red) neurons in selected subregions of the isocortex. Included are subregions with >2% labeling of excitatory inputs or >1.5% labeling of outputs. Significant differences in excitatory inputs between BFCN_→ACA_ and BFCN_→BLA_ neurons are indicated by asterisks. *P < 0.05; **P < 0.01; ***P < 0.001; *t* test. Data are presented as the means ± SEM. (**G** to **J**) Similar to (A) to (D) but for comparisons among inhibitory-input and output networks between BFCN_→ACA_ and BFCN_→BLA_ neurons. (**K**) Similar to (F) but for inhibitory inputs (solid bars) and outputs (empty bars) of BFCN_→ACA_ (green) and BFCN_→BLA_ (red) neurons in selected subregions of the STR. Included are subregions with >2% labeling of inhibitory inputs or >1.5% labeling of outputs. (**L**) Similar to (K) but for selected subregions of the PAL and the HY. (**M**) Diagrams illustrating the long-range reciprocal input-output loop between the BFCN_→ACA_ and BFCN_→BLA_ networks.

A high positive CC (0.86) between the excitatory-input networks of the two BFCN subtypes suggests shared excitatory-input sources (Fig. 8A and figs. S15 and S16). For example, the isocortex provides excitatory inputs to both BFCN subtypes, 29% for BFCN_→ACA_ neurons and 34% for BFCN_→BLA_ neurons (Fig. 8B). Analyzing output and excitatory-input networks across BFCN subtypes revealed distinct interaction patterns: A low CC of −0.4 between the BFCN_→BLA_ output and BFCN_→ACA_ excitatory-input networks indicates minimal interactions, suggesting that these networks likely operate independently (Fig. 8C). Conversely, a high positive CC of 0.89 between the BFCN_→ACA_ output and BFCN_→BLA_ excitatory-input networks indicates that BFCN_→ACA_ neurons modulate the excitatory inputs to BFCN_→BLA_ neurons by connecting to their excitatory-input sources (Fig. 8D).

Our data also show that BFCN_→ACA_ neurons provide extensive projections to the isocortex, which is a shared excitatory input source for both BFCN subtypes. The prefrontal cortices received the largest amount of cholinergic projections from BFCN_→ACA_ neurons, accounting for 48% of their cortical projections, and provided the largest amount of cortical excitatory inputs to both BFCN subtypes (BFCN_→ACA_, 50%; BFCN_→BLA_, 57%) (Fig. 8E). Notably, compared to BFCN_→ACA_ neurons, BFCN_→BLA_ neurons receive significantly higher proportions of excitatory inputs from the PL and AI (Fig. 8F). Given the established role of these cortical areas in top-down modulation of amygdalar activity during emotion processing (*61*, *68*, *69*), the preferential excitation of BFCN_→BLA_ neurons by these areas may enhance top-down control for cholinergic modulation of emotion.

We subsequently analyzed the interactions between the inhibitory-input networks of the BFCN subtypes. A high positive CC of 0.99 between their inhibitory-input networks again suggests shared inhibitory-input sources (Fig. 8, G and H). For example, the STR, HY, and PAL provide inhibitory inputs to both BFCN subtypes (Fig. 8H). Analyzing the output and inhibitory-input networks across BFCN subtypes revealed distinct interaction patterns. A low CC of −0.14 between the BFCN_→ACA_ output and BFCN_→BLA_ inhibitory-input networks indicates minimal interaction, suggesting that these networks likely operate independently (Fig. 8I). In contrast, a high positive CC (0.60) between the BFCN_→BLA_ output and BFCN_→ACA_ inhibitory-input networks indicates that BFCN_→BLA_ neurons apparently modulate the inhibitory inputs to BFCN_→ACA_ neurons by connecting to their inhibitory input sources (Fig. 8J).

BFCN_→BLA_ neurons provide extensive projections to shared inhibitory-input sources such as the STR and PAL. BFCN_→BLA_ neurons modulate BFCN_→ACA_ inhibitory inputs by targeting the CP and CeA in the STR and by targeting the SI in the PAL (Fig. 8, K and L). The CP provides significantly more inhibitory inputs to BFCN_→ACA_ neurons than to BFCN_→BLA_ neurons, whereas the CeA and SI provide more to BFCN_→BLA_ neurons (Fig. 8, K and L). We also noted that several subregions that preferentially inhibit BFCN_→ACA_ or BFCN_→BLA_ neurons receive few cholinergic projections from either BFCN subtype (e.g., the NAc and LS of the STR, and the MS and BST of the PAL). The LS and MS preferentially inhibit BFCN_→ACA_ neurons, while the NAc and BST preferentially inhibit BFCN_→BLA_ neurons (Fig. 8, K and L).

Given the established roles of the CP, LS, and MS in modulating cognitive functions and brain states, including decision-making, learning, memory, and arousal (*1*, *5*, *62*, *63*, *70*–*73*), their preferential inhibition of BFCN_→ACA_ neurons may facilitate brain-state–related cholinergic modulation of cognition. Moreover, given the known roles of the NAc, CeA, and BST in emotion processing (*61*, *74*–*78*), their preferential inhibition of BFCN_→BLA_ neurons may facilitate the cholinergic modulation of emotion. In addition, in light of the SI’s known involvement in regulating cognitive functions and brain states (*79*, *80*), its preferential inhibition of BFCN_→BLA_ neurons may enhance the interaction between the cholinergic modulation of cognition and emotion.

Together, our input-output network analyses collectively delineate that BFCN_→ACA_ and BFCN_→BLA_ neurons exhibit distinct roles in shaping each other’s excitatory-input and inhibitory-input networks: BFCN_→ACA_ neurons target a shared excitatory-input source, while BFCN_→BLA_ neurons target shared inhibitory-input sources, establishing a long-range reciprocal input-output loop (diagram shown in Fig. 8M). This loop supports dynamic interactions among the BFCN subtypes by enabling cholinergic modulation of both excitatory and inhibitory inputs.

## DISCUSSION

In this study, we identified two distinct subtypes of BFCNs (BFCN_→ACA_ and BFCN_→BLA_ neurons) and delineated their whole-brain input-output networks, characterized by input neuron type specificity. Both subtypes share inhibitory inputs from the STR, HY, and PAL, yet they have different networks for excitatory inputs and outputs. BFCN_→ACA_ neurons are activated by subcortical excitatory inputs from the MB and HY, providing broad cholinergic modulation to the isocortex, and receive excitatory feedback from the cortical areas that they innervate. In contrast, BFCN_→BLA_ neurons are activated by cortical and thalamic excitatory inputs, providing cholinergic modulation to the STR, CTXsp, and PAL, and receive excitatory feedback from the CTXsp and inhibitory feedback from the STR and PAL. Ultimately, our study found a long-range reciprocal input-output loop between these two BFCN subtypes: BFCN_→ACA_ neurons target a shared excitatory-input source—the isocortex, while BFCN_→BLA_ neurons target shared inhibitory-input sources—the STR and PAL, thus enabling cholinergic modulation of excitatory and inhibitory inputs among BFCN subtypes to facilitate their dynamic interactions.

BFCNs that share common targets are organized into distinct yet overlapping pools, and adjacent BFCNs may project to distinct downstream targets (*1*, *5*, *21*, *24*, *81*). Our identification of two distinct subtypes—BFCN_→ACA_ and BFCN_→BLA_ neurons—and the quantitative analyses of their somatic and axonal distributions support this notion. Our data reveal that BFCN_→ACA_ and BFCN_→BLA_ neurons form separate pools across multiple subregions of the PAL. Despite their somas being intermingled within each subregion, these BFCNs exhibit distinct projection patterns throughout the brain. BFCN_→ACA_ neurons target the isocortex, whereas BFCN_→BLA_ neurons target subcortical areas such as the STR, CTXsp, and PAL. Given the known functions of the prefrontal cortices in cognition processing and of the amygdala in emotion processing (*82*–*86*), our findings that BFCN_→ACA_ and BFCN_→BLA_ neurons, respectively, target these areas suggests their distinct roles in the cholinergic modulation of cognition and emotion.

An individual BFCN typically innervates regions that are physically distant but functionally related (*1*, *5*, *21*, *22*). ACh release in prefrontal cortices enhances detection of sensory cues during goal-oriented behaviors, thereby facilitating goal-driven attention (*7*, *8*, *87*), while ACh release in sensory cortices increases response reliability to sensory stimuli, enhancing stimulus-driven attention (*52*, *88*). BFCN_→ACA_ neurons, which innervate prefrontal, somatosensory, and visual cortices, are thus well-positioned to coordinately modulate both goal- and stimulus-driven attention.

Previous studies have reported that rostrally located BFCNs innervate both superficial and deep cortical layers, whereas caudally located BFCNs innervate deep layers (*1*, *81*, *89*). Notably, we observed that BFCN_→ACA_ neurons innervate both superficial and deep layers of the prefrontal cortices; further, BFCN_→ACA_ neurons preferentially innervate deep layers of the somatosensory and visual cortices. Our results suggest that rostrally located BFCN_→ACA_ neurons selectively innervate prefrontal cortices and that caudally located BFCN_→ACA_ neurons innervate both prefrontal and sensory cortices. This would represent an intriguing layer-specific organization for cholinergic modulation of cortical functions across hierarchical levels, potentially for top-down attention modulation of sensory processing.

ACh release in the BLA contributes to the encoding of emotionally salient memories (*11*–*13*). BFCN_→BLA_ neurons also innervate the CeA, which is involved in the expression of emotional responses such as freezing in response to fear (*78*, *90*–*92*). This innervation pattern may allow BFCN_→BLA_ neurons to coordinately modulate the processing of emotional memories and the expression of emotions.

The cholinergic system exerts diverse functions across a variety of temporal scales, including “tonic” (seconds to hours) and “phasic” (subsecond) effects (*93*–*95*). For example, regular spiking of BFCNs contributes to tonic effects, such as sustained attention and modulation of brain states (*53*). In contrast, transient burst spiking of BFCNs contributes to phasic effects, including the rapid modulation of attention to salient stimuli such as detecting rewards and punishments (*79*). Recall that for both the BFCN_→ACA_ and BFCN_→BLA_ populations, we observed subpopulations exhibiting regular spiking (NASP-BFCNs) or transient spiking (SLN-BFCNs). This apparent dual mechanism for ACh modulation in theory would allow the brain to balance (i) baseline cognitive and emotional functions with (ii) the ability to rapidly adapt to new information. Notably, there are significantly more transient spiking BFCN_→BLA_ than BFCN_→ACA_ neurons, suggesting greater phasic cholinergic modulation in emotion processing, presumably facilitating quick reactions to threats.

Our study supports previous findings about the distribution of BFCN inputs (*24*–*26*) while also providing additional insights into specific input neuron types. Those three studies identified the STR, HY, and PAL as major input sources for BFCNs; our findings reveal that both BFCN subtypes receive more inhibitory than excitatory inputs, with the STR, HY, and PAL being major sources of these inhibitory inputs. Moreover, our study highlights differences in inhibitory inputs to the two BFCN subtypes within the STR and PAL. For example, the CP and LS preferentially inhibit BFCN_→ACA_ neurons, whereas the NAc, CeA, and BST preferentially inhibit BFCN_→BLA_ neurons. Considering the well-established roles of the CP and LS in regulating cognitive functions and brain states—such as decision-making, learning, memory, and arousal (*62*, *71*–*73*)—their preferential inhibition of BFCN_→ACA_ neurons likely supports brain state–dependent cholinergic modulation of cognition. Similarly, given the recognized involvement of the NAc, CeA, and BST in emotion processing (*61*, *74*–*78*), their preferential inhibition of BFCN_→BLA_ neurons may contribute to cholinergic modulation of emotion processing. Note that the MS and NDB of the PAL provides more inhibitory and excitatory inputs to BFCN_→ACA_ neurons than to BFCN_→BLA_ neurons. Given the known roles of the MS and NDB in memory formation, attention, cognitive flexibility, arousal, and brain-state regulation (*1*, *5*, *63*), their preferential interaction with BFCN_→ACA_ neurons likely also supports brain state–dependent cholinergic modulation of cognition.

Previous studies have reported the existence of inhibitory long-range projections from the HPF to the BF (*96*), but any functions of these projections remain unknown. Our study reveals that the CA of the HPF, a subregion composed of ~80% excitatory neurons and ~20% inhibitory neurons (*28*–*30*), preferentially excites BFCN_→BLA_ neurons and preferentially inhibits BFCN_→ACA_ neurons. Given the known roles of the CA in memory expression (*97*, *98*), this distinct input pattern from the CA to BFCN_→ACA_ and BFCN_→BLA_ neurons could contribute to the differential shaping of cholinergic modulation of cognition and emotion based on memory context.

Expanding beyond this, our study found distinct excitatory-input sources for each BFCN subtype. Contrary to aforementioned reports of sparse putative excitatory inputs from the isocortex, we observed large amounts of excitatory inputs to both BFCN subtypes from the isocortex. This discrepancy is likely due to the use of the RV CVS-N2cΔG strain (*54*) in our study, which offers an order of magnitude enhancement in transsynaptic transfer efficiency compared to the RV SAD-B19ΔG strain used previously. Among the 11 major brain regions, the isocortex provides the largest proportion of excitatory inputs to BFCN_→BLA_ neurons, with the prefrontal cortical areas contributing 57% of these cortical inputs, suggesting that BFCN_→BLA_ neurons integrate cognition-related information to modulate emotion processing.

Notably, in addition to regulating BLA activity through the activation of BFCN_→BLA_ neurons, the prefrontal cortical areas also directly innervate the BLA (*99*). For example, the PL and ILA provide strong, topographically organized projections to the BLA (*99*). Note that PL→BLA projections have been previously functionally linked to fear expression and that ILA→BLA projections have been linked to fear extinction (*61*, *83*, *100*). In contrast, projections from the ACA to the BLA are relatively sparse; these have been functionally linked with value-based decision-making (*42*, *83*, *101*). Thus, the prefrontal cortical areas apparently exert cognitive control over emotion processing via direct glutamatergic inputs to the BLA and via indirect cholinergic inputs through activation of BFCN_→BLA_ neurons.

Our study also revealed differences in the subcortical regions providing excitatory inputs to BFCN subtypes. For example, we found that the MB and HY provide more excitatory inputs to BFCN_→ACA_ neurons than to BFCN_→BLA_ neurons. Owing to the mixed distribution of excitatory and inhibitory neurons reported in the MB and HY (*28*–*30*), previous studies have been unable to pinpoint the specific types of input neurons originating from these regions. By distinguishing between input neuron types in our study, we were able to attribute excitatory inputs from these regions, specifically to BFCN_→ACA_ neurons. Notably, the total number of excitatory input neurons in the MB and HY surpasses the total number of such neurons in the isocortex. Given the well-established roles of the MB and HY in global brain-state regulation and emotion processing (*61*, *64*, *65*, *67*, *71*, *102*), our findings suggest that BFCN_→ACA_ neurons integrate subcortical brain state– and emotion-related information to modulate cognition.

Benefiting from the Allen Mouse Brain Atlas (*56*), we obtained quantitative matrices representing the distribution of inputs and outputs across the brain for both BFCN subtypes, enabling analyses of their distinct network relationships at various hierarchical levels. For example, with BFCN_→ACA_ neurons, the high positive CC (0.76) between the major regions in the excitatory-input and output networks indicates preferential reciprocal connections with excitatory input sources. In addition, the even higher CC (0.89) between their excitatory-input and output networks within isocortex subregions suggests that cortical areas receiving more extensive cholinergic projections from BFCN_→ACA_ neurons also provide more excitatory feedback to these neurons.

On the basis of the specific connection patterns in the input-output networks of each BFCN subtype and the long-range reciprocal loop between them, we propose a working model for the interaction between cholinergic modulation of cognition and of emotion: BFCN_→ACA_ neurons are activated by brain state– and emotion-related subcortical excitatory inputs, providing cholinergic modulation of cognition by targeting the isocortex, especially the prefrontal cortices. The prefrontal cortices in turn provide cognition-related excitatory cortical inputs to activate BFCN_→BLA_ neurons, which modulate emotion processing by targeting amygdalar subregions. In addition, BFCN_→BLA_ neurons innervate shared subcortical inhibitory input sources, which provide brain state– and emotion-related inhibition to both BFCN subtypes.

Our anatomical characterization of the input-output networks, tailored to the specific types of input neurons for these two distinct BFCN subtypes, sheds light on the neural architecture underlying the cholinergic modulation of cognition and emotion. This study lays a foundation for future physiological investigations into the coordinated, cholinergic modulation–based regulation of cognition and emotion processing.

While our study provides insights into the organization of input-output networks of distinct BFCN subtypes, several limitations should be considered. First, RV tracing with triple-plex RNAscope in situ hybridization supports mapping of the distributions of excitatory and inhibitory inputs (*Vglut^+^* and *Vgat^+^* input neurons) to specific BFCN subtypes; however, this approach does not yield information about synaptic properties (e.g., synaptic strength and dynamics) that would be needed to characterize the function(s) of a specific input. It should be possible to explore, in behaviorally relevant contexts, how diverse excitatory and inhibitory inputs from various regions differentially influence BFCN subtypes, as well as how in vitro electrophysiological properties may translate into in vivo functional outcomes. Such evidence would likely clarify how information flows in the input-output networks of BFCN subtypes. Second, our cross-correlation analyses for input-output networks of BFCNs revealed potentially informative relationships between inputs and outputs occurring at multiple hierarchical levels, but these data are insufficient for demonstrating functional relationships between input and output regions of the two populations of BFCNs. Such relationships could be further investigated using for example in vitro and in vivo electrophysiological recordings. Last, our analysis did not identify significant differences related to age or gender, so the possibility of nuanced effects cannot be ruled out. Larger sample sizes could help assess whether these factors influence the organization and function of BF cholinergic circuits.

## MATERIALS AND METHODS

### Animals

Animal care and the experimental protocols were approved by the Animal Committee of Shanghai Jiao Tong University School of Medicine and the Animal Committee of the Institute of Neuroscience, Chinese Academy of Sciences. ChAT-Cre mice (Jackson, #006410) were obtained from the Jackson Laboratory. Male and female mice aged 5 to 24 weeks were used (table S7). Mice were housed in 12-hour light-dark cycle (lights on at 07:00 a.m. and off at 07:00 p.m.) with free access to food and water.

### Virus

The Retro-AAVs Retro-AAV-EF1α-DIO-EGFP (genomic titer, 5 × 10^12^ gc/ml) and Retro-AAV-EF1α-DIO-mCherry (5 × 10^12^ gc/ml) were acquired from BrainCase. The AAVs AAV-hSyn-EGFP (2 × 10^12^ gc/ml) and AAV-hSyn-mCherry (5 × 10^12^ gc/ml) were acquired from BrainCase. AAV9-EF1α-DIO-TVA-mCherry (6 × 10^12^ gc/ml) and AAV9-EF1α-DIO-N2cG (3 × 10^12^ gc/ml) were acquired from BrainVTA. Glycoprotein-deleted (ΔG) and EnvA-pseudotyped RV (CVS-N2cΔG-EGFP+EnvA, 2 × 10^8^ IU/ml) was acquired from BrainVTA.

### Surgery

Adult mice were anesthetized with isoflurane (5% induction and 1.5% maintenance) and placed on a stereotaxic frame (Ruiwode Life Science). The temperature was kept at 37°C throughout the procedure using a heating pad. After asepsis, the skin was incised to expose the skull and the overlying connective tissue was removed. A craniotomy (~0.5-mm diameter) was made above the injection site. Viruses were loaded in a sharp micropipette mounted on a Nanoject II attached to a micromanipulator and then injected at a speed of 60 nl per min. Coordinates used were as follows: ACA (bregma, +0.3 mm; lateral, 0.3 mm; and depth, 0.9 mm), BLA (bregma −1.8 mm, lateral 3.3 mm, and depth 3.8 mm), MO (bregma +0.3 mm, lateral 1.2 mm, and depth 1.0 mm), and BF (bregma 0.0 mm, lateral 1.4 mm, and depth 4.9 mm).

To examine the distribution and electrophysiological properties of BFCN_→ACA_ and BFCN_→BLA_ neurons, we injected Cre-inducible Retro-AAV expressing EGFP (Retro-AAV-EF1α-DIO-EGFP, 400 nl) in the ACA, and Cre-inducible Retro-AAV expressing mCherry (Retro-AAV-EF1α-DIO-mCherry, 100 to 150 nl) in the BLA of ChAT-Cre mice, respectively. To examine the injection site in the ACA, we coinjected Retro-AAV-EF1α-DIO-EGFP and AAV9-hSyn-EGFP (1:1, 400 nl) in some experiments. To examine the injection site in the BLA, we coinjected Retro-AAV-EF1α-DIO-mCherry and AAV9-hSyn-mCherry (1:1, 100 to 150 nl) in some experiments. In a control experiment, we reversed the virus injections between the ACA and BLA. Specifically, Retro-AAV-EF1α-DIO-EGFP and AAV9-hSyn-EGFP (1:1, 100 to 150 nl) were injected in the BLA, while Retro-AAV-EF1α-DIO-mCherry and AAV9-hSyn-mCherry (1:1, 400 nl) were injected in the ACA. The histology experiments and brain slice recording experiments were performed 2 weeks after virus injection.

To examine the distribution of BFCN_→ACA_ neurons and BFCN_→MO_ neurons, we coinjected Retro-AAV-EF1α-DIO-EGFP and AAV9-hSyn-EGFP (1:1, 400 nl) in the ACA, and coinjected Retro-AAV-EF1α-DIO-mCherry and AAV9-hSyn-mCherry (1:1, 400 nl) in the MO of ChAT-Cre mice. The histology experiments were performed 2 weeks after virus injection.

In a control experiment, we varied both the amount and type of Retro-AAV injected in the BLA of ChAT-Cre mice to assess the consistency of the distribution of retrogradely labeled BFCN_→BLA_ neurons. The following conditions were tested: (i) 150 nl, Retro-AAV-EF1α-DIO-EGFP and AAV9-hSyn-EGFP (1:1); (ii) 100 nl, Retro-AAV-EF1α-DIO-mCherry and AAV9-hSyn-mCherry (1:1); and (iii) 150 nl, Retro-AAV-EF1α-DIO-mCherry and AAV9-hSyn-mCherry (1:1). The histology experiments were performed 2 weeks after virus injection.

To examine the whole-brain axon distribution patterns of BFCN_→ACA_ and BFCN_→BLA_ neurons, we injected Cre-inducible AAV expressing TVA receptors (AAV9-EF1α-DIO-TVA-mCherry, 200 nl) in the BF of ChAT-Cre mice. Two weeks later, CVS-N2cΔG-EGFP+EnvA was injected in the ACA (300 nl) or the BLA (100 nl). The histology experiments were performed 7 days after RV injection.

For retrograde monosynaptic tracing from BFCN_→ACA_ and BFCN_→BLA_ neurons, we coinjected AAV9-EF1α-DIO-TVA-mCherry and AAV9-EF1α-DIO-N2cG (1:2, 300 nl) in the BF of ChAT-Cre mice. Two weeks later, CVS-N2cΔG-EGFP+EnvA was injected in the ACA (300 nl) or the BLA (100 nl). In a control experiment, we coinjected AAV9-hSyn-mCherry and CVS-N2cΔG-EGFP+EnvA (1:1, 300 nl) in the ACA to verify the injection site. The histology experiments were performed 7 days after RV injection.

To examine potential TVA leak expression in non-Cre neurons, we injected AAV-EF1α-DIO-TVA-mCherry (200 nl) in the BF of wild-type mice. Two weeks later, CVS-N2cΔG-EGFP+EnvA was injected into the ACA (300 nl) or BLA (100 nl). The histology experiments were performed 7 days after RV injection.

### Slice preparation and recording

Slice preparation and recording followed precedures described previously (*35*, *38*, *80*, *103*). Mice were anesthetized with 5% isoflurane. After decapitation, the brain was dissected rapidly and placed in ice-cold oxygenated *N*-methyl-d-aspartate (NMDG)–Hepes solution [in millimolar: NMDG, 93; KCl, 2.5; NaH_2_PO_4_, 1.2; NaHCO_3_, 30; Hepes, 20; glucose, 25; sodium ascorbate, 5; thiourea, 2; sodium pyruvate, 3; MgSO_4_.7H_2_O, 10; CaCl_2_.2H_2_O, 0.5; and NAC, 12 (at pH 7.4), adjusted with HCl], and coronal sections of brain slices were made with a vibratome. Slices (300 μm thick) were recovered in oxygenated NMDG-Hepes solution at 32°C for 10 min and then maintained in an incubation chamber with oxygenated standard ACSF (in millimolar: NaCl, 125; KCl, 3; CaCl_2_, 2; MgCl_2_, 1; NaH_2_PO_4_, 1.25; sodium ascorbate, 1.3; NaHCO_3_, 26; and glucose, 10) at 30°C for 1 to 4 hours before recording.

Whole-cell recordings were made at 30°C in oxygenated standard ACSF. EPSPs were recorded using a potassium-based internal solution [in millimolar: K-gluconate, 135; KCl, 5; Hepes, 10; EGTA, 0.3; MgATP, 4; Na_2_GTP, 0.3; and Na_2_-phosphocreatine 10 (at pH 7.3), adjusted with KOH, 290 to 300 mosmol]. The resistance of the patch pipette was 3 to 5 megohm. The cells were excluded if the series resistance exceeded 40 megohm or varied by more than 20% during the recording period. Data were recorded with a Multiclamp 700B amplifier (Axon instruments) filtered at 2 kHz and digitized with a Digidata 1322 (Axon instruments) at 10 kHz. Recordings were analyzed using custom software.

### Histology

Mice were deeply anesthetized with isoflurane and immediately perfused with chilled 0.1 M phosphate-buffered saline (PBS) followed by 4% paraformaldehyde (w/v) in PBS. The brain was removed and postfixed overnight at 4°C. After fixation, the brain was placed in 30% sucrose (w/v) in PBS solution for 1 to 2 days at 4°C. After embedding and freezing, the brain was sectioned into 50-μm coronal slices using a cryostat. However, for RNAscope experiments, the brain was sectioned into 25-μm coronal slices.

For fluorescence images without staining, brain slices were washed with PBS for 30 min and mounted with VECTASHIELD mounting medium with 4′,6-diamidino-2-phenylindole (DAPI). To examine the distribution of BFCN_→ACA_ and BFCN_→BLA_ neurons, every third brain slices (50-μm) were imaged in the high-throughput slide scanners (VS120, Olympus) for further processing. To reveal the distribution of starter cells in RV-assisted input mapping, every sixth section (25-μm) was imaged without staining (VS120, Olympus). We also imaged selected example slices under a confocal microscope (Nikon TiE-A1 plus).

#### Tyramide signal amplification

Alexa Fluor 488 Tyramide SuperBoost Kits (goat anti-rabbit IgG, B40922, Thermo Fisher Scientific) were used to enhance the EGFP signal in thin axons. Brain slices were rehydrated with PBS for 30 min, followed by a 10-min incubation in 3% H_2_O_2_. After another 10-min wash with PBS, the slices were permeabilized using PBST (0.3% Triton X-100 in PBS) for 10 min. The slices were then incubated for 2 hours in blocking solution (5% normal goat serum in PBST), followed by an overnight incubation at 4°C with anti-GFP rabbit polyclonal antibody (A-11122, Thermo Fisher Scientific; dilution 1:500). The following day, the slices were washed five times with PBST and incubated with poly–horseradish peroxidase (HRP)–conjugated secondary antibody (goat anti-rabbit, the Tyramide SuperBoost Kits) for 1 hour at 37°C. After three PBS washes, the slices were treated with Tyramide working solution for 5 min at room temperature and then with Reaction Stop Reagent. Following three additional PBS washes, the slices were mounted with VECTASHIELD mounting medium containing DAPI (H-2000, Vectorlabs). Every third section was imaged using high-throughput slide scanners (VS120, Olympus) for further processing.

#### *Triple-plex RNAScope* in situ *hybridization*

RNAScope Fluorescent Multiplex Reagent Kit (322000, 322381, 323110, 323110, and 322809, ACDBio) was used to identify excitatory and inhibitory input neurons for BFCN_→ACA_ and BFCN_→BLA_ neurons.

##### Day 1

Brain slices were first rehydrated in PBS for 10 min and then incubated with 3% H_2_O_2_ for 10 min, followed by a 1-min wash with diethyl pyrocarbonate (DEPC) solution (0.1% DEPC in ddH_2_O). The slices underwent heat retrieval in boiling retrieval solution for 6 min and were then washed again with DEPC solution for 1 min. Subsequently, the slices were incubated twice with 100% ethanol for 2 min each and left air-dry at room temperature overnight.

##### Day 2

Protease III was applied to the slices, which were then incubated for 30 min at 40°C. Subsequently, the slices were washed twice with DEPC solution for 5 min each. For hybridization, the slices were incubated with probes for Vgat, Vglut1, Vglut2, and EGFP (Slc32a1-C1, Slc17a7-C2, Slc17a6-C2, and EGFP-C3), diluted at a ratio of 50:1:1:1, for 2 hours at 40°C in a HybEZ humidified incubator. After hybridization, the slices were rinsed twice with ACD wash buffer for 2 min each. To amplify the signals, the slices were successively incubated with AMP1-FL, AMP2-FL, and AMP3-FL solutions for 30, 30, and 15 min, respectively, at 40°C, each followed by two 2-min rinses with wash buffer.

To develop the HRP-C1 signal, the slices were incubated with HRP-C1 for 15 min at 40°C, rinsed twice with wash buffer for 2 min each, and subsequently incubated with TSA Plus Cy3 (dilution 1:1000 in TSA buffer) for 30 min at 40°C. This was followed by two 2-min rinses with wash buffer. The slices were then treated with HRP blocker for 15 min at 40°C and rinsed twice with wash buffer for 2 min each. These steps were repeated to develop the HRP-C2 and HRP-C3 signals. Specifically, for HRP-C2 signal development, the slices were sequentially incubated with HRP-C2 and TSA Plus Cy5 (dilution 1:1000 in TSA buffer). For HRP-C3 signal development, the slices underwent sequential incubation with HRP-C3 and TSA Plus fluorescein (dilution 1:1000 in TSA buffer). Last, the slices were mounted with VECTASHIELD mounting medium containing DAPI. Every sixth section was imaged using high-throughput slide scanners (VS120, Olympus) for further analysis.

### 3D reconstruction and quantification

A custom-written software package was used to process the digitized brain images. The analysis software consists of four modules: atlas rotation, image registration, signal detection, and quantification/visualization. The detailed method has been described previously (*25*, *35*, *37*).

#### Rotation module

The rotation module facilitates the 3D rotation of the Allen Mouse Brain Atlas by arbitrary angles to align it with each sample. Anatomical landmarks were manually selected to determine the rotation angles. To estimate the rotation angle about the left-right axis, the most posterior slices displaying the CA3 in each hemisphere were used. For the dorsal-ventral axis, the rotation angle was estimated using the most anterior slice that shows the anterior commissure crossing the midline and the most posterior slice where the corpus callosum crosses the midline. The reference atlas was then rotated on the basis of these estimated angles to mimic the aberrant sectioning angle of the experimental brain.

#### Registration module

The registration module is a reference point-based image alignment software used to align images of brain sections with the rotated 3D reference atlas for subsequent quantification and 3D reconstruction. Initially, reference points were chosen in both the atlas and the brain image. The module then performed geometric transformations on the brain section to optimize the alignment of these reference points between the brain image and the atlas. After this transformation, the alignment between the image and the atlas was inspected. Further manual adjustments were made as necessary to ensure precise matching.

#### Detection module

The detection module comprises two independent submodules designed for counting cells and detecting axons, respectively. The cell counting module logs the positions of manually identified labeled neurons within each digitized brain section image. For axon detection, a ridge detection method was used (http://en.wikipedia.org/wiki/Ridge_detection). The process to enhance detection accuracy involves several steps:

(i) Computation of image ridges: Ridges are computed across multiple scales to extract all potential axon-like signals from each image, resulting in a binary “ridge image.” In this image, the number of pixels representing each detected axon correlates with its length rather than thickness. However, this ridge image also contains many noise pixels alongside valid axons.

(ii) Noise reduction based on background intensity: To eliminate noise pixels stemming from general background fluorescence, a threshold is set on the basis of the intensity distribution of the original image. This threshold is used as a mask to filter out noise pixels from the ridge image produced in the first step.

(iii) Removal of discrete high-intensity noise pixels: Discrete noise pixels with fluorescence intensities higher than the general background are removed by setting a threshold for spatially contiguous pixels. Pixels that are spatially contiguous in the ridge image are identified, the size of each contiguous region is calculated, and regions below a predetermined size threshold are removed. This step is repeated along with the previous noise reduction step until satisfactory detection results are achieved.

(iv) Visual inspection and manual correction: The final step involves a visual inspection of the results. The remaining noise pixels, primarily artifacts introduced during brain tissue processing, are manually discarded.

#### Quantification/visualization module

After detection and registration, signals were quantified across the whole brain and projected to the 3D reference atlas for better visualization.

### Quantification and statistical analysis

#### Distribution of labeled neurons and axons

Given that the number of labeled neurons varied across brains, and aiming to assess an equally weighted population average for each brain, we calculated the distribution percentage of labeled BFCNs in each PAL subregion by dividing the number of labeled BFCNs in that region by the total number of labeled neurons in the PAL.

For the distribution of BFCN axons (i.e., the cholinergic output), the axon projection to each region was quantified as the number of pixels occupied by detected axons in the cleaned ridge image divided by the total number of axon-occupied pixels detected in the entire brain.

Besides the input neurons labeled solely with *Vglut*^+^ or *Vgat*^+^ signal, we also observed a small population of input neurons labeled with both signals (1%) or with neither of these two signals (8%); such neurons were excluded from any subsequent calculations. The “excitatory input percentage” from each region was quantified by dividing the number of *Vglut*^+^ input neurons found in that region by the total number of *Vglut*^+^ input neurons in the whole brain. Similarly, the “inhibitory input percentage” was quantified by dividing the number of *Vgat*^+^ input neurons in each region by the total number of *Vgat*^+^ input neurons. The E/(E + I) ratio of input neurons in each region was calculated by dividing the number of *Vglut*^+^ input neurons by the total of *Vglut*^+^ and *Vgat^+^* input neurons found in the same region. Similarly, the I/(E + I) ratio of input neurons in each region was calculated by dividing the number of *Vgat*^+^ input neurons by the total of *Vglut*^+^ and *Vgat^+^* input neurons found in the same region.

#### Statistical analysis

All statistical tests and data analysis were performed using MATLAB and Graphpad Prism. All statistical tests were two-sided. The exact number of mice and recorded cells were described in figure legends. Data were expressed as means ± SEMs in figures and text. Statistical method, statistics, and corresponding *P* values were reported in the figure legends.
