# Supplementary material for: Whole-brain mapping of basal forebrain cholinergic neurons reveals a long-range reciprocal input-output loop between distinct subtypes
Source: Sci Adv. 2025 May 30;11(22):eadt1617. doi: 10.1126/sciadv.adt1617 (PMC12124396; doi:10.1126/sciadv.adt1617)
Supplement: Supplementary file 1 — Figs. S1 to S16 Legends for tables S1 to S7 Legends for movies S1 to S4 [file sciadv.adt1617_sm.pdf]

Science Advances
AAAS

Supplementary Materials for

Whole-brain mapping of basal forebrain cholinergic neurons reveals a long-range reciprocal input-output loop between distinct subtypes

Zhaonan Chen *et al.*

Corresponding author: Min Xu, mxu@ion.ac.cn; Siyu Zhang, zhang_siyu@sjtu.edu.cn

Sci. Adv. **11**, eadt1617 (2025)
DOI: 10.1126/sciadv.adt1617

The PDF file includes:

Figs. S1 to S16
Legends for tables S1 to S7
Legends for movies S1 to S4

Other Supplementary Material for this manuscript includes the following:

Tables S1 to S7
Movies S1 to S4

SUPPLEMENTARY FIGURES AND FIGURE CAPTIONS

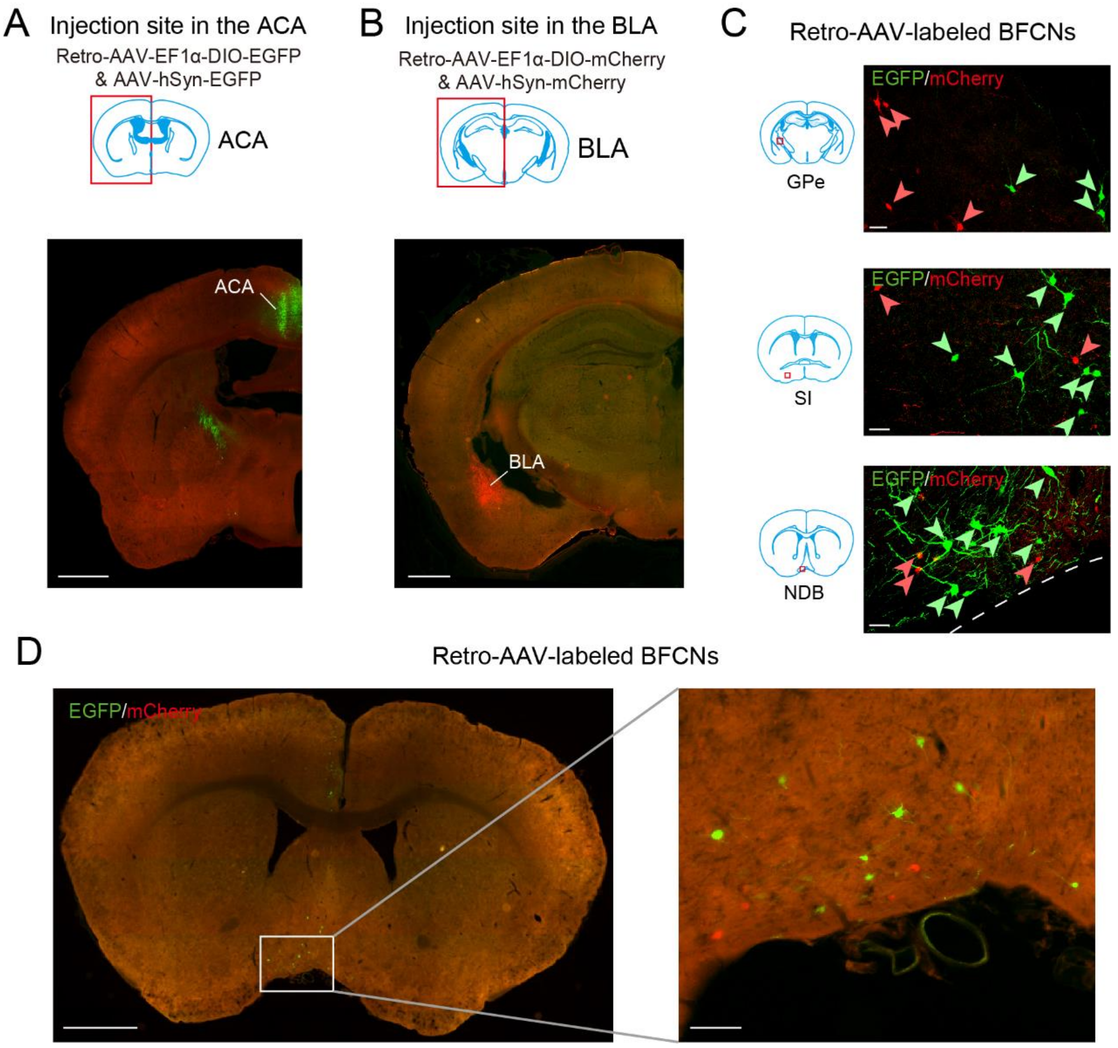

fig. S1. Injection sites of Retro-AAV in the ACA and BLA, and retrogradely labeled BFCNs in various BF subregions.

(A) Fluorescence image showing the injection site of Retro-AAV-EF1α-DIO-EGFP and AAV-hSyn-EGFP in the ACA (green) of ChAT-Cre mice. Scale bar, 1 mm.

(B) Fluorescence image showing the injection site of Retro-AAV-EF1α-DIO-mCherry and AAV-hSyn-mCherry in the BLA (red) of ChAT-Cre mice. Scale bar, 1 mm.

(C) Confocal fluorescence images showing the Retro-AAV-labeled BFCNs in the GPe, SI, and NDB (red boxes in coronal diagrams). Green, EGFP; red, mCherry. Green arrowheads indicate $BFCN_{\rightarrow ACA}$ neurons, and red arrowheads indicate $BFCN_{\rightarrow BLA}$ neurons. Scale bar, 50 µm.

(D) Fluorescence images acquired with a VS200 microscope. Left, image showing Retro-AAV-labeled $BFCN_{\rightarrow ACA}$ (green) and $BFCN_{\rightarrow BLA}$ (red) neurons. Scale bar, 1 mm. Right, zoomed-in view of the white rectangle. Scale bar, 100 μm.

Relevant abbreviations: ACA, anterior cingulate cortex; BLA, basolateral amygdala; GPe, external globus pallidus; SI, substantia innominata; NDB, diagonal band nucleus.

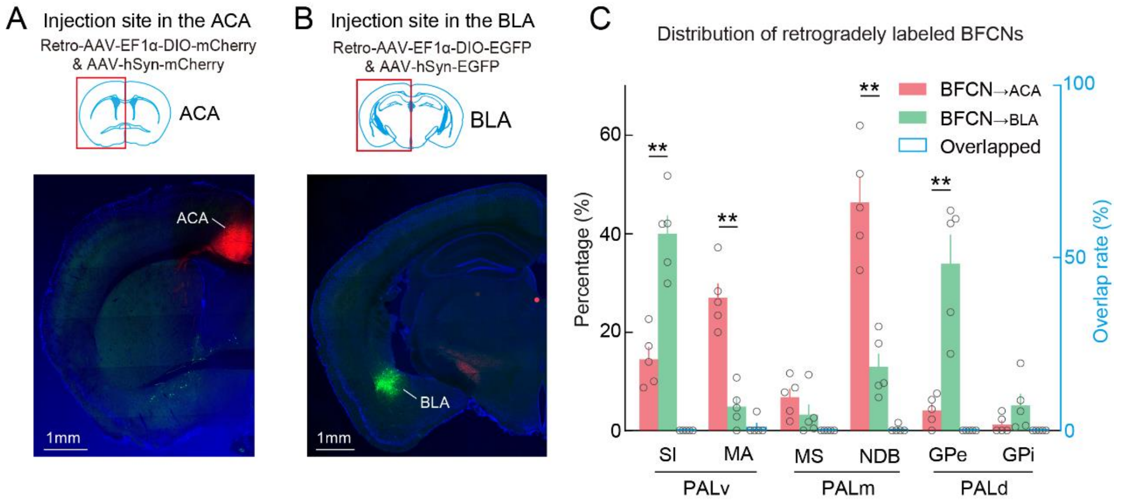

fig. S2. Exchanging Retro-AAV injections between the ACA and BLA resulted in a similar distribution of retrogradely labeled BFCN$_{\rightarrow ACA}$ and BFCN$_{\rightarrow BLA}$ neurons compared to before the exchange.

(A) Fluorescence image showing the injection site of Retro-AAV-EF1α-DIO-mCherry and AAV-hSyn-mCherry in the ACA (red) of ChAT-Cre mice. Scale bar, 1 mm.

(B) Fluorescence image showing the injection site of Retro-AAV-EF1α-DIO-EGFP and AAV-hSyn-EGFP in the BLA (green) of ChAT-Cre mice. Scale bar, 1 mm.

(C) Distribution of retrogradely labeled BFCN$_{\rightarrow ACA}$ (red bar) and BFCN$_{\rightarrow BLA}$ (green bar) neurons (n = 5 mice). The distribution differs significantly across PAL subregions and BFCN subtypes ($F_{region}(5,4) = 195$, $P_{region} = 7 \times 10^{-5}$, $F_{region*type}(5,4) = 91$, $P_{region*type} = 3 \times 10^{-4}$, two-way mixed ANOVA). There are significantly more BFCN$_{\rightarrow BLA}$ than BFCN$_{\rightarrow ACA}$ neurons in the SI and GPe (SI, P = 0.009; GPe, P = 0.009, Tukey's HSD test). In contrast, there are significantly more BFCN$_{\rightarrow ACA}$ than BFCN$_{\rightarrow BLA}$ neurons in the MA and NDB (MA, P = 0.004; NDB, P = 0.01). Notably, there was little overlap (blue bar) among the PAL subregions. Significant differences in the distribution between BFCN$_{\rightarrow ACA}$ and BFCN$_{\rightarrow BLA}$ neurons are indicated by asterisks. *, $P < 0.05$; **, $P < 0.01$. Data are presented as the mean ± SEM.

Relevant abbreviations: ACA, anterior cingulate cortex; BLA, basolateral amygdala; GPe, external globus pallidus; GPi, internal globus pallidus; SI, substantia innominata; MA, magnocellular nucleus; NDB, diagonal band nucleus; MS, medial septal nucleus.

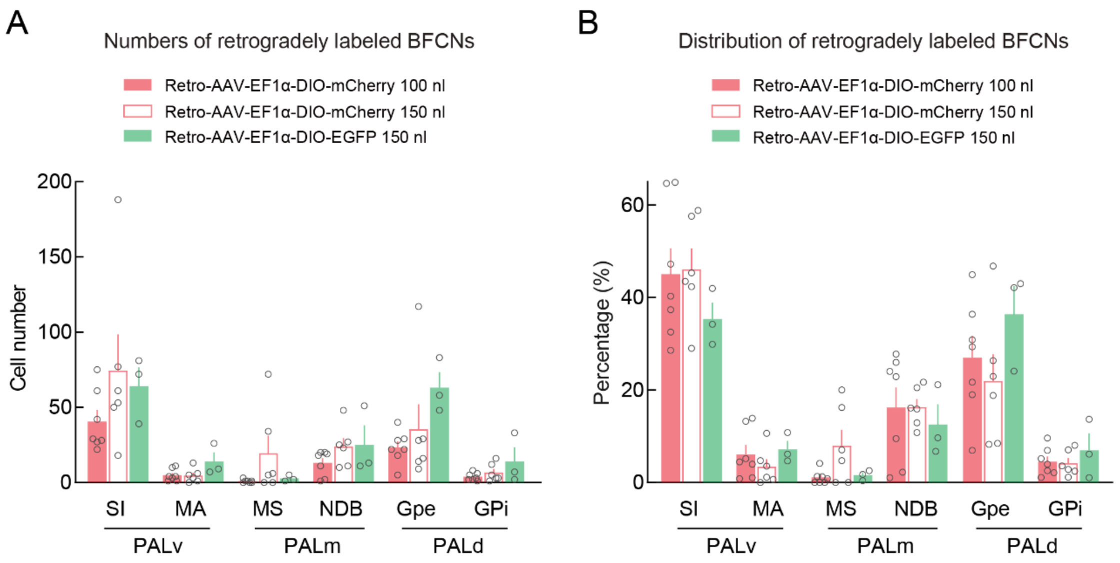

fig. S3. Numbers and proportions of Retro-AAV-labeled BFCN$_{\rightarrow BLA}$ neurons.

(A) The number of Retro-AAV-labeled BFCN$_{\rightarrow BLA}$ neurons in the indicated PAL subregions following varying volumes and types of viral vector injection. Data are presented as the mean ± SEM.

(B) The proportion of Retro-AAV-labeled BFCN$_{\rightarrow BLA}$ neurons in the indicated PAL subregions following varying volumes and types of viral vector injection.

Relevant abbreviations: GPe, external globus pallidus; GPi, internal globus pallidus; SI, substantia innominata; MA, magnocellular nucleus; NDB, diagonal band nucleus; MS, medial septal nucleus.

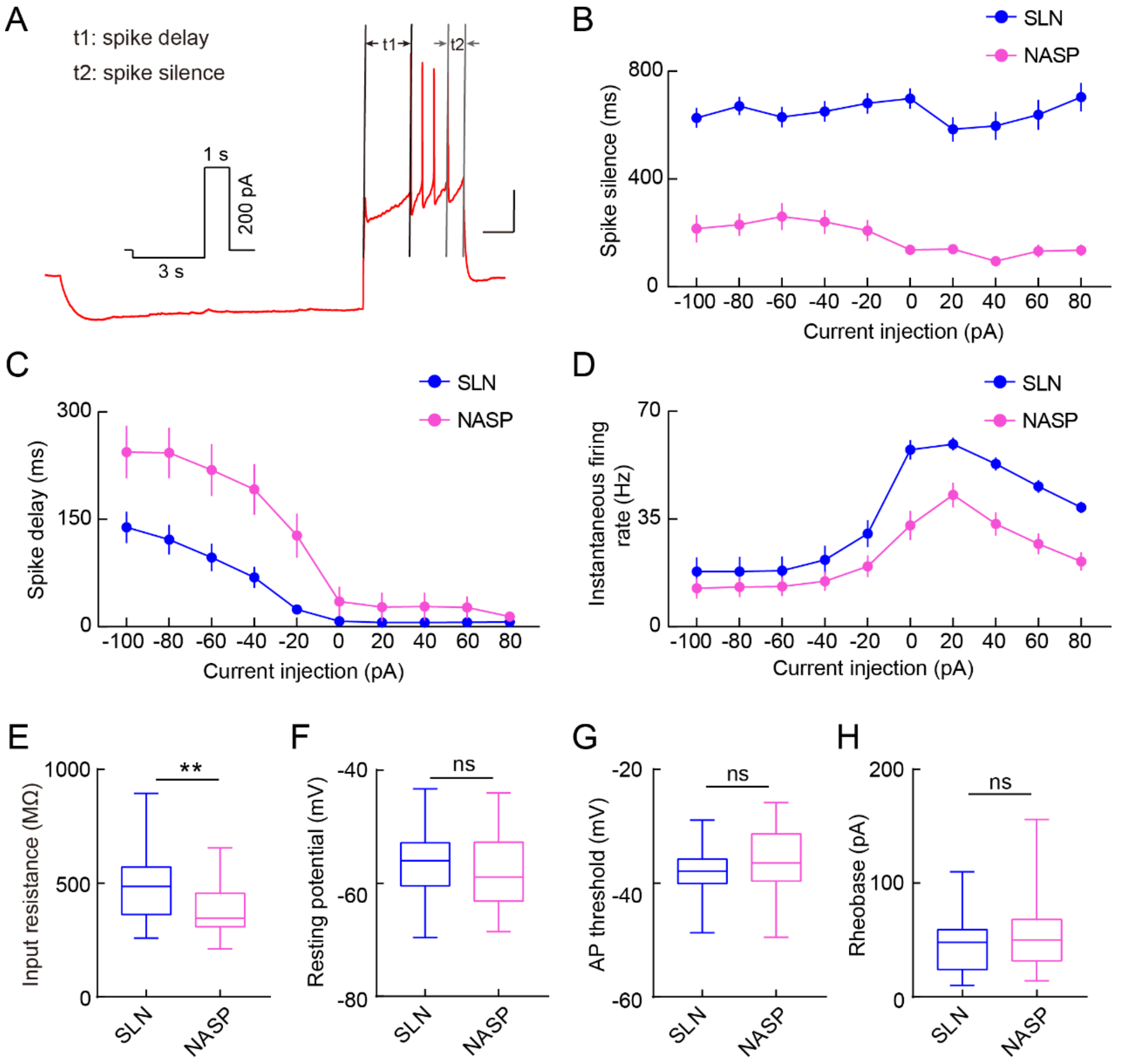

fig. S4. Firing Patterns and passive electrophysiological properties of NASP- and SLN-BFCNs.

(A) Example trace illustrating the firing pattern of a non-adapting spiking (NASP) neuron responding to a -20-pA pre-hyperpolarization followed by a 200-pA depolarization current injection. Scale bars, 300 ms, 20 mV. Inset, the current step used to generate this trace. Spike delay (t1), from the onset of the 200-pA current injection to the start of the first spike during this injection. Spike silence (t2), from the peak of the last spike during the 200-pA current injection to the end of this injection.

(B) NASP-BFCNs showed significantly shorter no-spiking periods (spike silence) compared to SLN-BFCNs during a 1-second suprathreshold current injection, unaffected by the prepolarization current level ($F_{cell\_type}(1,640) = 598.2$, $P_{cell\_type} = 9 \times$

10^{-94}; $F_{current\_level}(9,640) = 1.5$, $P_{cell\_type} = 0.13$; $F_{cell\_type*current\_level}(9,640) = 1.2$, $P_{cell\_type} = 0.32$, two-way ANOVA; For current injection levels from -100 pA to 80 pA, NASP vs. SLN, $P < 4 \times 10^{-8}$, *t*-test). Data are presented as the mean ± SEM.

(C) Under hyperpolarized conditions, NASP-BFCNs showed significantly longer spike delays than SLN-BFCNs, a difference that was not observed under depolarized conditions ($F_{cell\_type}(1,640) = 62.7$, $P_{cell\_type} = 1 \times 10^{-14}$; $F_{current\_level}(9,640) = 31.7$, $P_{cell\_type} = 4 \times 10^{-46}$; $F_{cell\_type*current\_level}(9,640) = 3.6$, $P_{cell\_type} = 2 \times 10^{-4}$, two-way ANOVA. For current injection levels from -100 pA to -20 pA, NASP vs. SLN, $P < 0.02$; from 0 pA to 80 pA, $P > 0.14$, *t*-test). Data are presented as the mean ± SEM.

(D) NASP-BFCNs had significantly lower instantaneous firing rates compared to SLN-BFCNs under depolarized conditions ($F_{cell\_type}(1,640) = 60.8$, $P_{cell\_type} = 3 \times 10^{-14}$; $F_{current\_level}(9,640) = 28.6$, $P_{cell\_type} = 7 \times 10^{-42}$; $F_{cell\_type*current\_level}(9,640) = 2.1$, $P_{cell\_type} = 0.03$, two-way ANOVA. For current injection levels from -100 pA to -20 pA, NASP vs. SLN, $P > 0.06$; from 0 pA to 80 pA, $P < 8 \times 10^{-4}$, *t*-test). The instantaneous firing rate was here defined as the firing rate calculated from the interval between the first two spikes during the 200-pA current injection. Data are presented as the mean ± SEM.

(E) Box plot for input resistances of SLN- and NASP-BFCNs. Edges, 25th and 75th percentiles; central line, median; whiskers, 1.5× the interquartile range of the edges. SLN-BFCNs exhibit significantly higher input resistance than NASP-BFCNs (SLN-BFCNs, n = 40 neurons; NASP-BFCNs, n = 26 neurons; $P = 0.002$, *t*-test). **, $P < 0.01$.

(F) Similar to (E), but for resting potential of SLN- and NASP-BFCNs ($P = 0.20$, *t*-test).

(G) Similar to (E), but for AP threshold of SLN- and NASP-BFCNs ($P = 0.11$, *t*-test).

(H) Similar to (E), but for rheobase of SLN- and NASP-BFCNs ($P = 0.25$, *t*-test).”

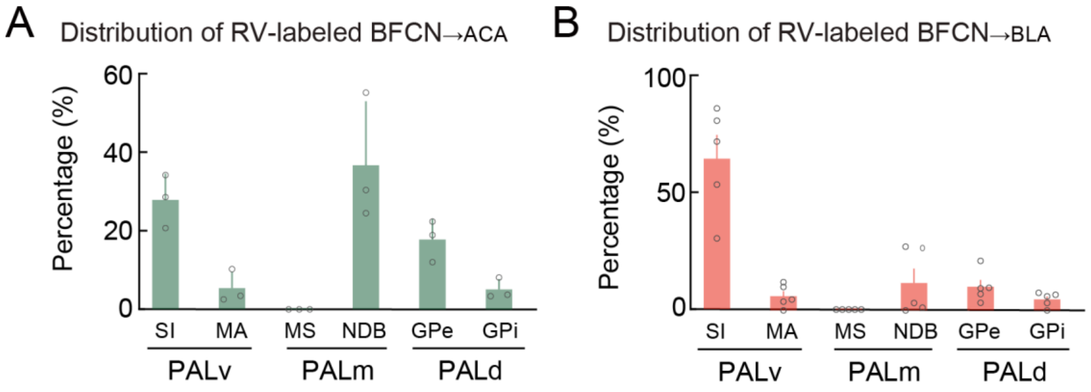

fig. S5. Distribution of RV-labeled BFCN$_{\rightarrow ACA}$ and BFCN$_{\rightarrow BLA}$ Neurons in Axon Mapping.

(A) Distribution of RV-labeled BFCN$_{\rightarrow ACA}$ neurons in the PAL (n = 3 mice). Data are presented as the mean ± SEM.

(B) Similar to (A), but for RV-labeled BFCN$_{\rightarrow BLA}$ neurons (n = 5 mice).

Relevant abbreviations: GPe, external globus pallidus; GPi, internal globus pallidus; SI, substantia innominata; MA, magnocellular nucleus; NDB, diagonal band nucleus; MS, medial septal nucleus.

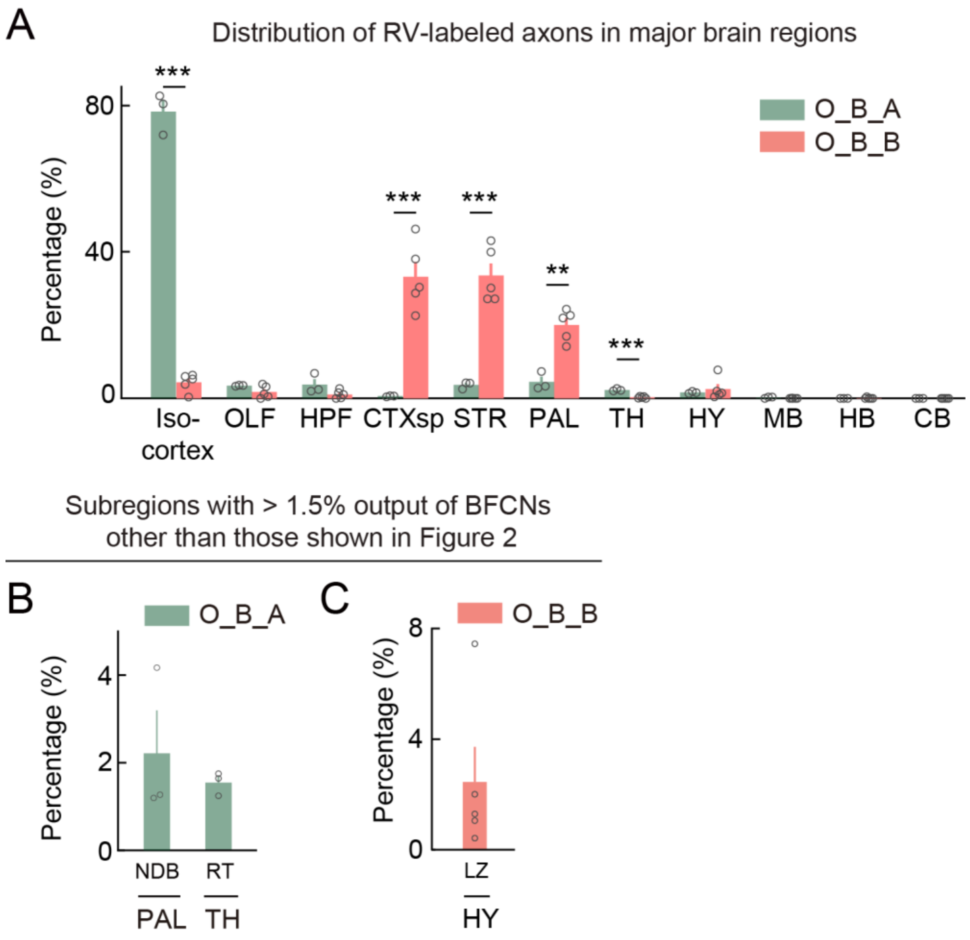

fig. S6. Distribution of RV-labeled Axons from BFCN$_{\rightarrow ACA}$ and BFCN$_{\rightarrow BLA}$ Neurons in Major Brain Regions and Subregions.

(A) Percentage of outputs for BFCN$_{\rightarrow ACA}$ (green) and BFCN$_{\rightarrow BLA}$ (red) neurons in major brain regions. Significant differences in outputs between BFCN$_{\rightarrow ACA}$ and BFCN$_{\rightarrow BLA}$ neurons are indicated by asterisks. **, $P < 0.01$; ***, $P < 0.001$; *t*-test. Data are presented as the mean ± SEM.

(B) Percentage of outputs for BFCN$_{\rightarrow ACA}$ neurons in the indicated subregions with > 1.5% output labeling (these two subregions were omitted from Figure 2 panel F). Data are presented as the mean ± SEM.

(C) Similar to (B), but for BFCN$_{\rightarrow BLA}$ neurons.

Relevant abbreviations: OLF, olfactory areas; HPF, hippocampal formation; CTXsp, cortical subplate; STR, striatum; PAL, pallidum; TH, thalamus; HY, hypothalamus; MB,

midbrain; HB, hindbrain; CB, cerebellum; NDB, diagonal band nucleus; RT, reticular nucleus of the thalamus; LZ, lateral hypothalamic zone.

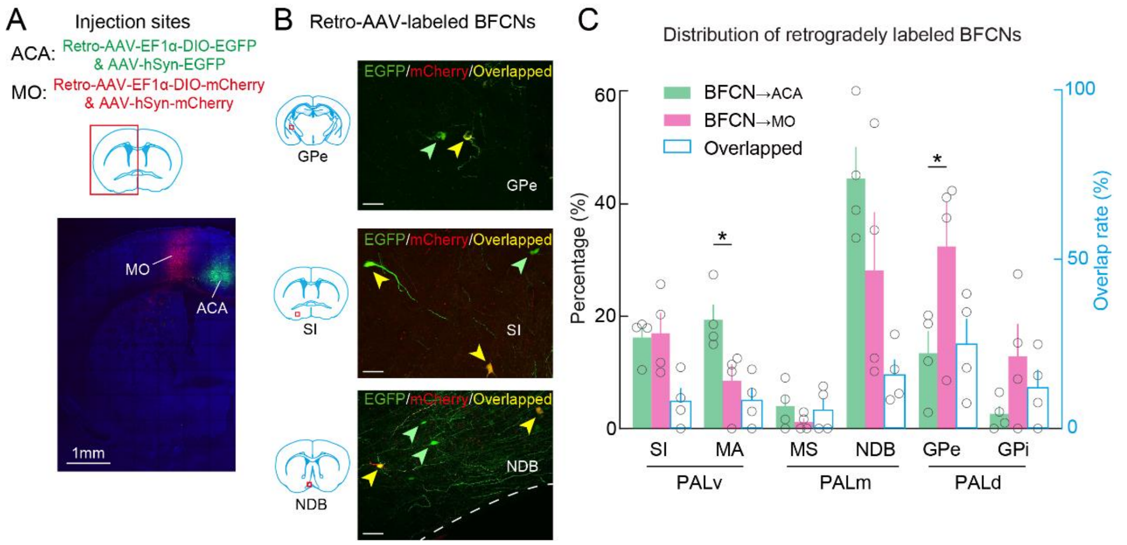

fig. S7. Distribution of BFCNs innervating the ACA and MO.

(A) Fluorescence image showing the injection sites in the ACA (green, Retro-AAV-EF1α-DIO-EGFP and AAV-hSyn-EGFP) and MO (red, Retro-AAV-EF1α-DIO-mCherry and AAV-hSyn-mCherry) of ChAT-Cre mice. Scale bar, 1 mm.

(B) Fluorescence images showing the Retro-AAV-labeled BFCNs in the GPe, SI, and NDB (red boxes in coronal diagrams). Green, EGFP; red, mCherry. Green arrowheads indicate $BFCN_{\rightarrow ACA}$ neurons; yellow arrowheads indicate BFCNs innervating both the ACA and MO. Scale bar, 50 μm.

(C) Distribution of retrogradely labeled $BFCN_{\rightarrow ACA}$ (green bar) and $BFCN_{\rightarrow MO}$ (pink bar) neurons (n = 4 mice). The distributions of $BFCN_{\rightarrow ACA}$ and $BFCN_{\rightarrow MO}$ neurons differ significantly across PAL subregions ($F_{region}(5,2) = 30$, $P_{region} = 0.03$, $F_{region*type}(5,2) = 4$, $P_{region*type} = 0.22$, two-way mixed ANOVA). There are significantly more $BFCN_{\rightarrow ACA}$ than $BFCN_{\rightarrow MO}$ neurons in the MA (P = 0.02, Tukey's HSD test). In contrast, there are significantly more $BFCN_{\rightarrow MO}$ than $BFCN_{\rightarrow ACA}$ neurons in the GPe (P = 0.03). Notably, the overlap rates of $BFCN_{\rightarrow ACA}$ and $BFCN_{\rightarrow MO}$ neurons range from 6% to 25% across PAL subregions. Significant differences in the distribution between $BFCN_{\rightarrow ACA}$ and $BFCN_{\rightarrow BLA}$ neurons are indicated by asterisks. *, $P < 0.05$. Data are presented as the mean ± SEM.

Relevant abbreviations: ACA, anterior cingulate cortex; MO, motor cortex; GPe, external globus pallidus; GPi, internal globus pallidus; SI, substantia innominata; MA, magnocellular nucleus; NDB, diagonal band nucleus; MS, medial septal nucleus.

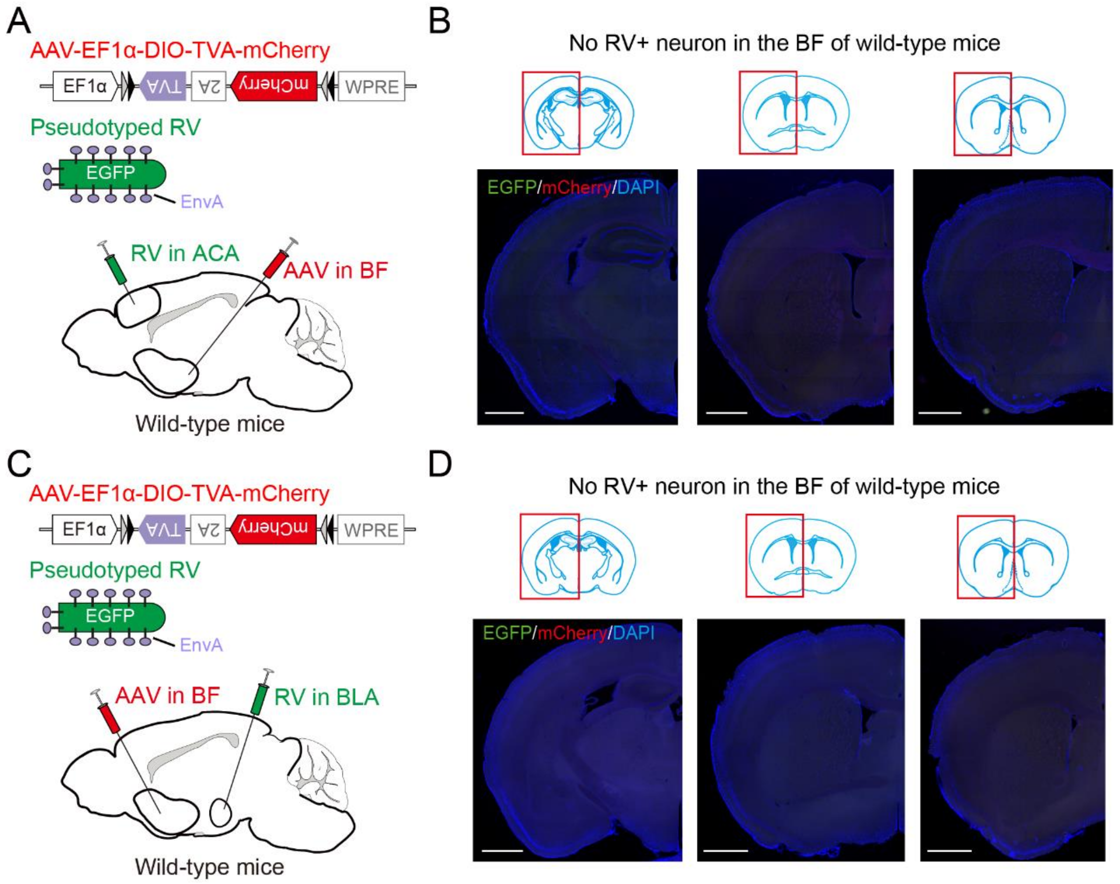

fig. S8. Control experiments for RV-assisted axon mapping.

(A) Viral vectors and injection procedure for a control experiment assessing RV-assisted axon mapping from BFCN$_{\rightarrow ACA}$ neurons.

(B) **N**o EGFP expression was observed upon injection of AAV-EF1α-DIO-TVA-mCherry in the BF followed by RV injection in the ACA of wild-type mice. Scale bar, 1 mm.

(C-D) Similar to (A-B) but for a control experiment of RV-assisted axon mapping from BFCN$_{\rightarrow BLA}$ neurons. **N**o EGFP expression was observed upon injection of AAV-EF1α-DIO-TVA-mCherry in the BF followed by RV injection in the BLA of wild-type mice. Scale bar, 1 mm.

A

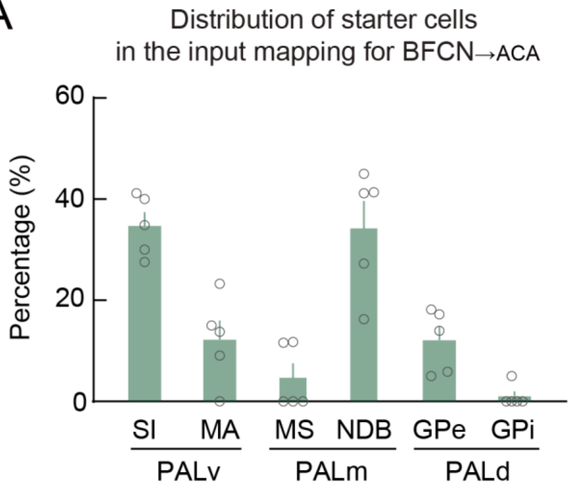

fig. S9. Distribution of Starter Cells in the Input Mapping for BFCN$_{\rightarrow ACA}$ Neurons.

(A) Distribution of starter cells in the PAL. Data are presented as the mean ± SEM, n = 5 mice.

Relevant abbreviations: GPe, external globus pallidus; GPi, internal globus pallidus; SI, substantia innominata; MA, magnocellular nucleus; NDB, diagonal band nucleus; MS, medial septal nucleus.

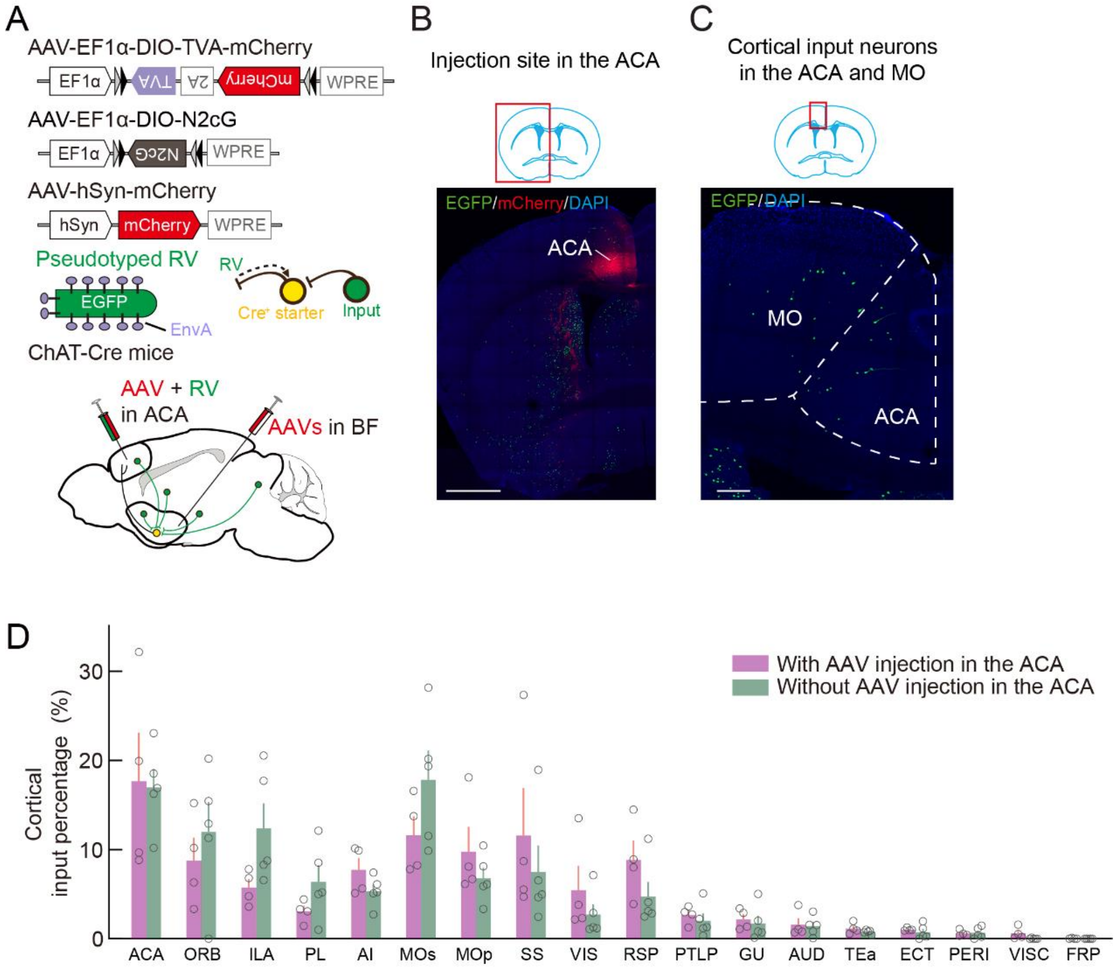

fig. S10. Verification of RV injection site in the ACA and the distribution of cortical input neurons.

(A) Viral vectors and injection procedure for RV-mediated trans-synaptic retrograde tracing from $BFCN_{\rightarrow ACA}$ neurons. Note that AAV-hSyn-mCherry was co-injected with RV into the ACA to examine the RV injection site.

(B) Fluorescence image showing RV and AAV injection site in the ACA (red). Scale bar, 1 mm. µm. Green, EGFP; red, mCherry; blue, DAPI.

(C) Fluorescence image showing retrogradely labeled input neurons (green) in the ACA and MO (red box in coronal diagram). Scale bar, 200 µm.

(D) Percentages of cortical inputs for $BFCN_{\rightarrow ACA}$ neurons with (purple) or without (green) AAV-hSyn-mCherry co-injection in the ACA in selected subregions of the isocortex. Included are subregions with >2% labeling of cortical inputs. No significant

differences in cortical inputs were found between these two groups. $P > 0.08$; *t*-test. Data are presented as the mean ± SEM.

Relevant abbreviations: ACA, anterior cingulate cortex; ORB, orbital cortex; ILA, infralimbic cortex; PL, prelimbic cortex; AI, agranular insular cortex; MOs, secondary motor cortex; MOp, primary motor cortex; SS, somatosensory cortex; VIS, visual cortex; RSP, retrosplenial cortex; PTLp, posterior parietal cortex; GU, gustatory cortex; AUD, auditory cortex; TEa, temporal association cortex; ECT, ectorhinal cortex; PERI, perihinal cortex; VISC, visceral cortex; FRP, frontal pole, cerebral cortex.

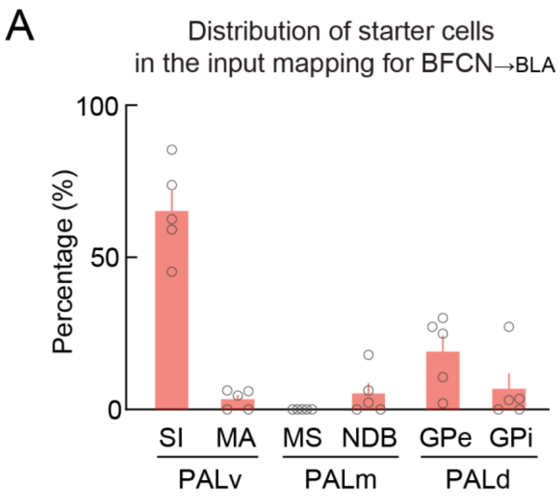

fig. S11. Distribution of Starter Cells in the Input Mapping for BFCN→BLA Neurons.

(A) Distribution of starter cells in the PAL. Data are presented as the mean ± SEM, n = 5 mice.

Relevant abbreviations: GPe, external globus pallidus; GPi, internal globus pallidus; SI, substantia innominata; MA, magnocellular nucleus; NDB, diagonal band nucleus; MS, medial septal nucleus.

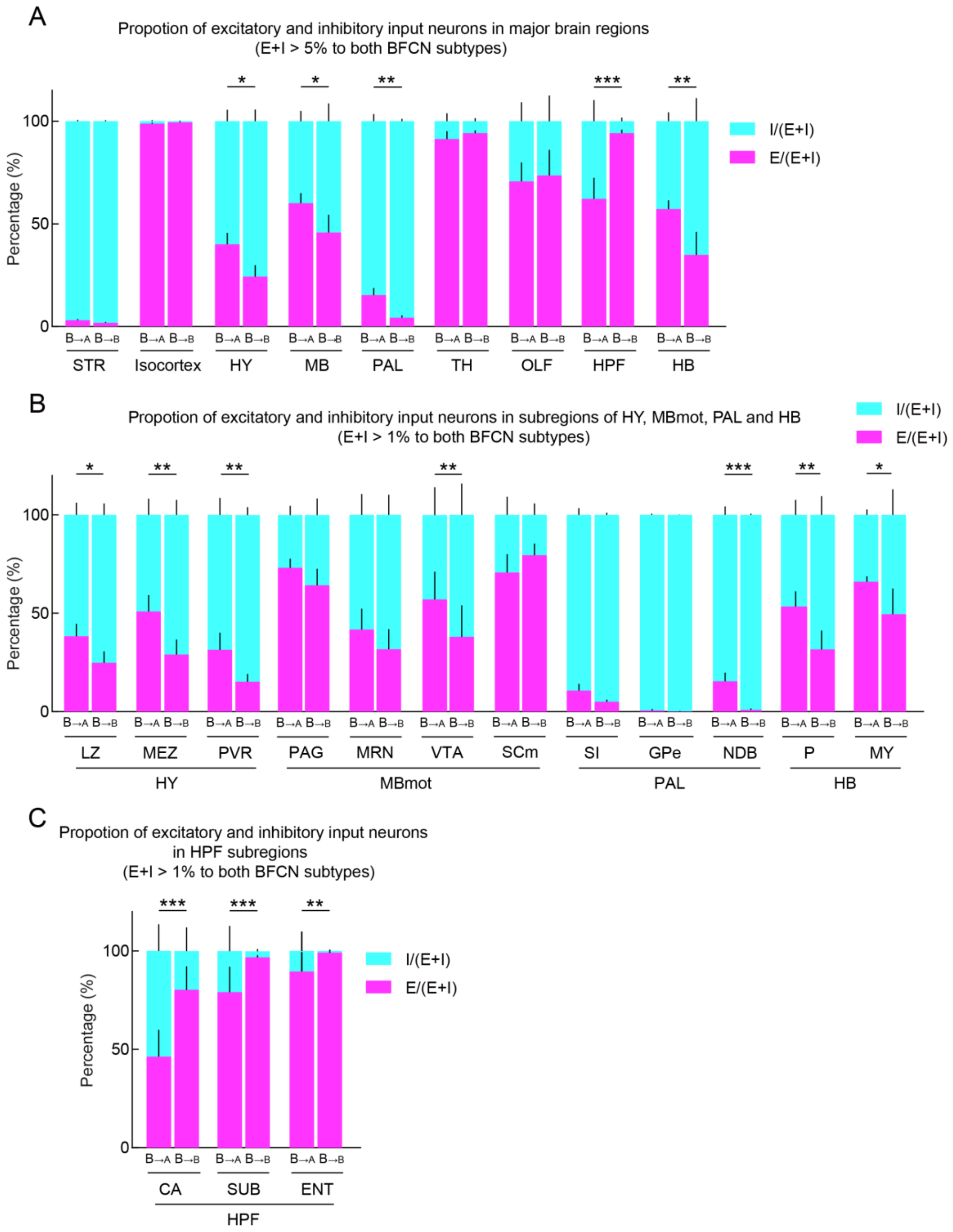

fig. S12. Proportion of excitatory and inhibitory input neurons in examined brain regions.

(A) Proportion of excitatory and inhibitory input neurons in the indicated examined major brain regions. Included are major regions with E+I >5% labeling for both BFCN$_{\rightarrow ACA}$ and BFCN$_{\rightarrow BLA}$ neurons. Data are presented as the mean ± SEM. *, P < 0.05; **, P < 0.01; ***, P < 0.001; chi-square test.

(B) Proportion of excitatory and inhibitory input neurons in the indicated subregions of the HY, MBmot, PAL, and HB. Included are subregions with E+I >1% labeling for both $BFCN_{\rightarrow ACA}$ and $BFCN_{\rightarrow BLA}$ neurons.

(C) Similar to (B), but for the indicated subregions of the HPF.

Relevant abbreviations: STR, striatum; HY, hypothalamus; MB, midbrain; PAL, pallidum; TH, thalamus; OLF, olfactory areas; HPF, hippocampal formation; HB, hindbrain; LZ, lateral hypothalamic zone; MEZ, medial hypothalamic zone; PVR, paraventricular nucleus; PAG, periaqueductal gray; MRN, midbrain reticular nucleus; VTA, ventral tegmental area; SCm, motor-related superior colliculus; SI, substantia innominata; NDB, diagonal band nucleus; GPe, external globus pallidus; P, pons; MY, medulla; CA, ammon's horn; SUB, subiculum; ENT, entorhinal area.

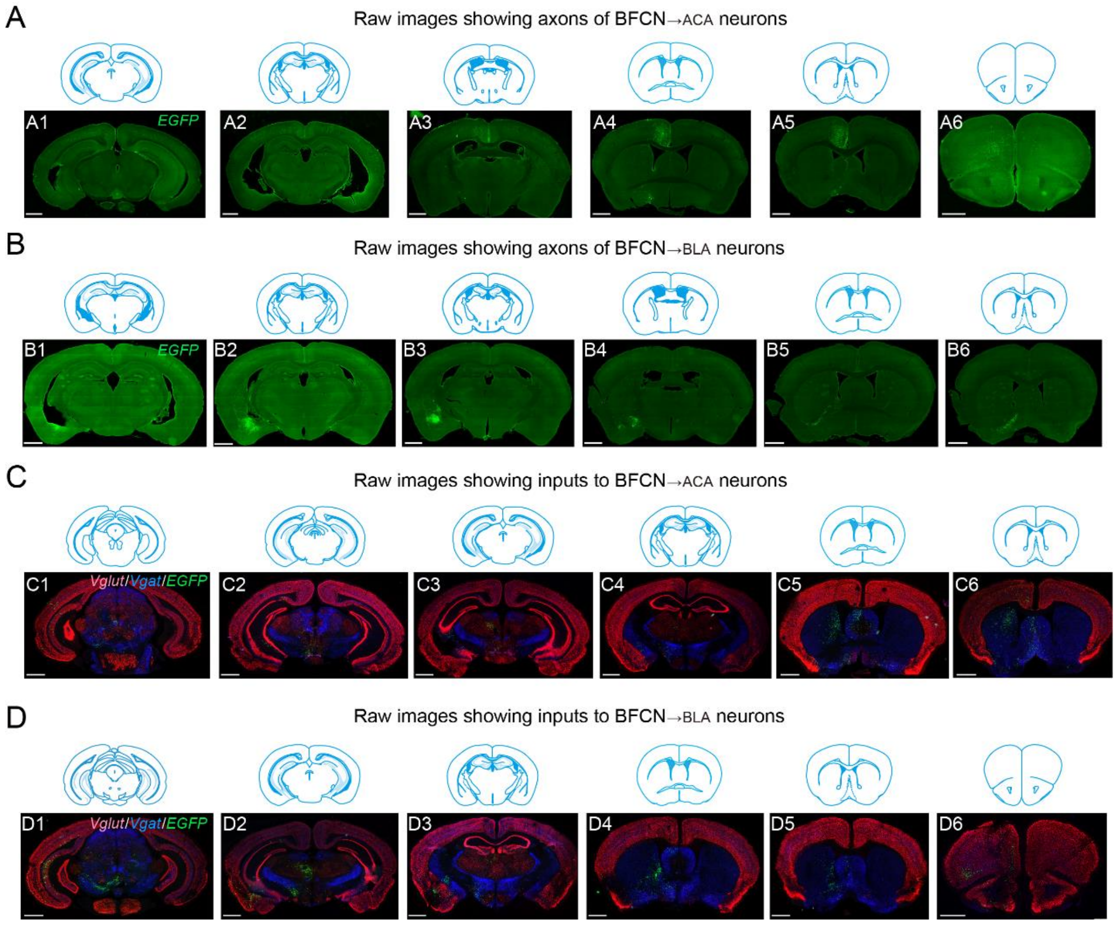

fig. S13. Raw images corresponding to digitized images in Figs. 2, 3, and 5.

(A) Raw images corresponding to digitized images in Fig. 2D, showing axons of $BFCN_{\rightarrow ACA}$ neurons (green). Green, EGFP. Scale bar, 1 mm.

(B) Similar to (A), but for Fig. 2J, showing axons of $BFCN_{\rightarrow BLA}$ neurons (green).

(C) Raw images corresponding to digitized images in Fig. 3D,E, showing inputs (green) to $BFCN_{\rightarrow ACA}$ neurons. Green, *EGFP*; red, *Vglut*; blue, *Vgat*. Scale bar, 1 mm.

(D) Similar to (C), but for Fig. 5D,E, showing inputs (green) to $BFCN_{\rightarrow BLA}$ neurons.

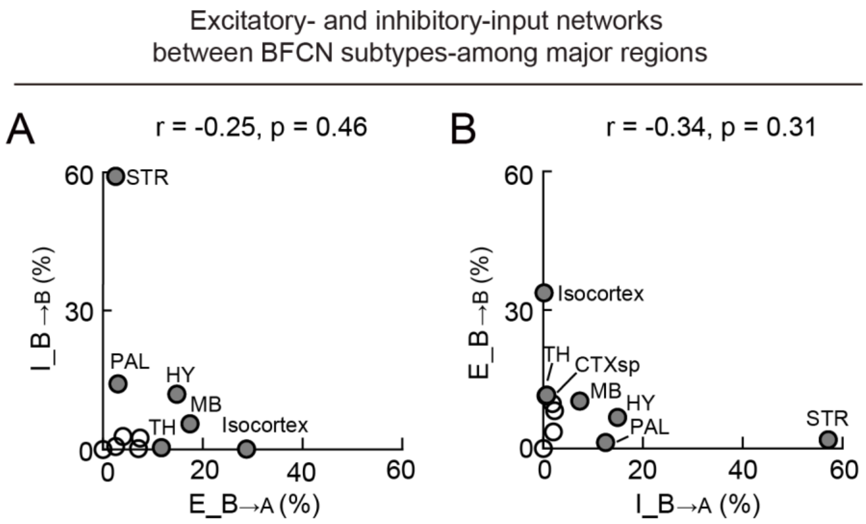

fig. S14. Minimal Interaction between the BFCN→ACA Excitatory-Input and BFCN→BLA Inhibitory-Input Networks, and *Vice Versa*.

(A) Inhibitory input percentages of BFCN→BLA neurons versus excitatory input percentages of BFCN→ACA neurons in major brain regions. Filled circles indicate strongly connected regions (with >10% labeling of excitatory or inhibitory inputs for either BFCN→BLA or BFCN→ACA neurons).

(B) Similar to (A), but for excitatory input percentages of BFCN→BLA neurons versus inhibitory input percentages of BFCN→ACA neurons.

Relevant abbreviations: PAL, pallidum; HY, hypothalamus; MB, midbrain; TH, thalamus; CTXsp, cortical subplate; STR, striatum.

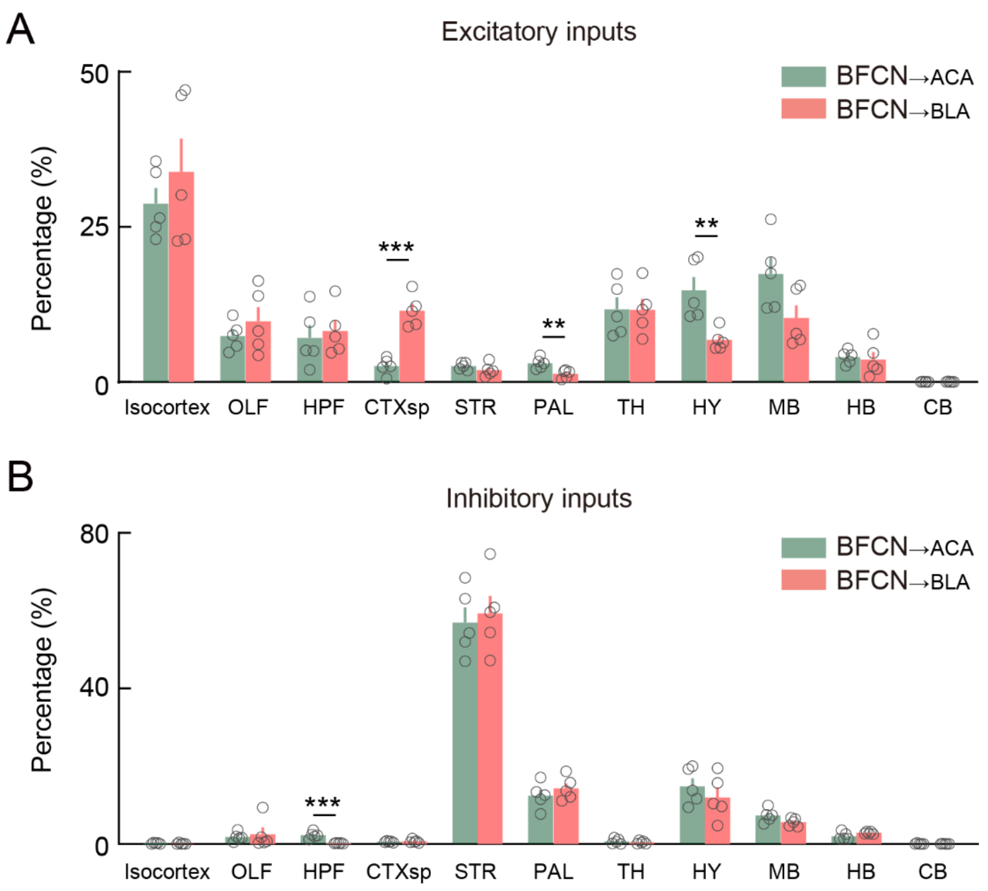

fig. S15. Distribution of Excitatory and Inhibitory Inputs of BFCN$_{\rightarrow ACA}$ and BFCN$_{\rightarrow BLA}$ Neurons in Major Brain Regions.

(A) Percentage of excitatory inputs of BFCN$_{\rightarrow ACA}$ (green) and BFCN$_{\rightarrow BLA}$ (red) neurons in major brain regions. Significant differences in excitatory inputs between BFCN$_{\rightarrow ACA}$ and BFCN$_{\rightarrow BLA}$ neurons are indicated by asterisks. **, $P < 0.01$; ***, $P < 0.001$; *t*-test. Data are presented as the mean ± SEM.

(B) Similar to (A), but for inhibitory inputs.

Relevant abbreviations: OLF, olfactory areas; HPF, hippocampal formation; CTXsp, cortical subplate; STR, striatum; PAL, pallidum; TH, thalamus; HY, hypothalamus; MB, midbrain; HB, hindbrain; CB, cerebellum.

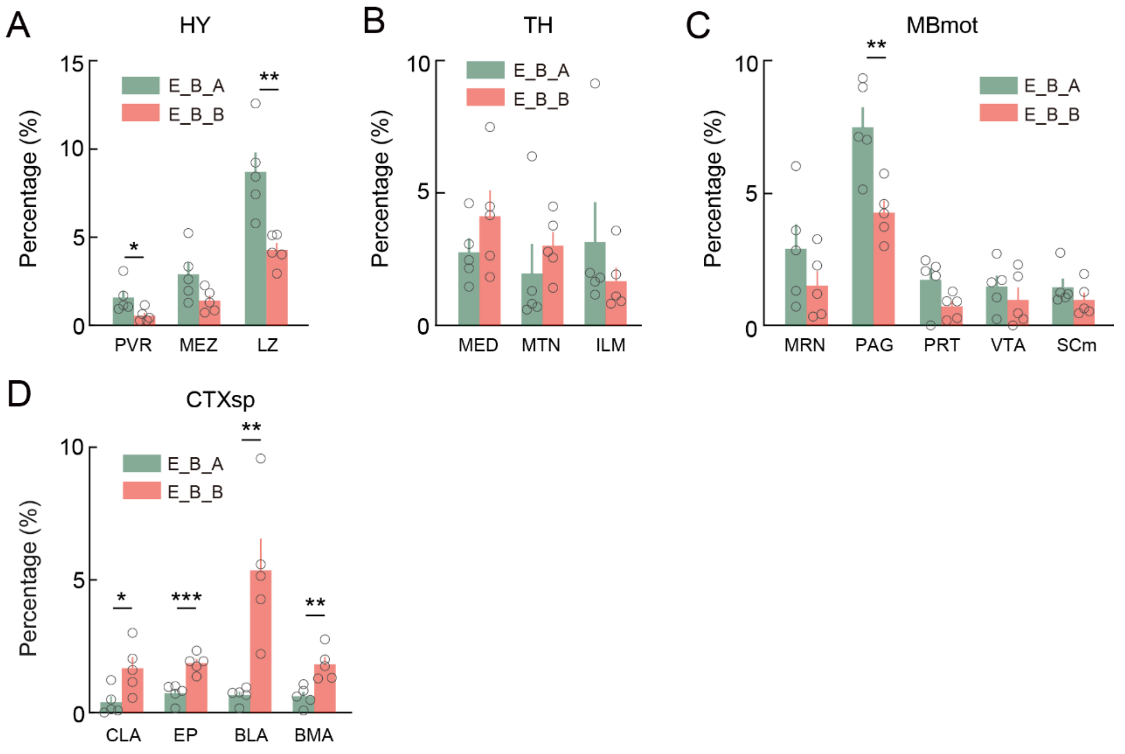

fig. S16. Distribution of Excitatory Inputs of BFCN→ACA and BFCN→BLA Neurons in Selected Subregions of the HY, TH, MBmot, and CTXsp.

(A) Percentage of excitatory inputs of BFCN→ACA (green) and BFCN→BLA (red) neurons in the indicated subregions of the HY. Included are subregions with >1% labeling of excitatory inputs. Data are presented as the mean ± SEM. Significant differences in excitatory inputs between BFCN→ACA and BFCN→BLA neurons are indicated by asterisks. *, $P < 0.05$; **, $P < 0.01$; ***, $P < 0.001$; *t*-test. Data are presented as the mean ± SEM.

(B) Similar to (A), but for selected subregions of the TH.

(C) Similar to (A), but for selected subareas of the MBmot.

(D) Similar to (A), but for selected subregions of the CTXsp.

Relevant abbreviations: HY, hypothalamus; TH, thalamus; MBmot, motor-related midbrain; CTXsp, cortical subplate; PVR, paraventricular nucleus; MEZ, medial hypothalamic zone; LZ, lateral hypothalamic zone; MED, medial group of the dorsal thalamus; MTN, midline group of the dorsal thalamus; ILM, intralaminar nuclei of the dorsal thalamus; MRN, midbrain reticular nucleus; PAG, periaqueductal gray; PRT, pretectal region; VTA, ventral tegmental area; SCm, motor-related superior colliculus;

CLA, claustrum; EP, endopiriform nucleus; BLA, basolateral amygdala; BMA, basomedial amygdala.

SUPPLEMENTARY TABLE LEGENDS

Table S1. Abbreviations of brain regions referenced in this study.

Table S2. Hierarchical organization of brain regions. Red indicates structures referenced in this study.

Table S3. Data summary for the distribution of Retro-AAV labeled BFCN$_{\rightarrow ACA}$ and BFCN$_{\rightarrow BLA}$ neurons in the PAL.

Table S4. Data summary for the whole-brain distribution of output from BFCN$_{\rightarrow ACA}$ and BFCN$_{\rightarrow BLA}$ neurons.

Table S5. Data summary for the whole-brain distribution of excitatory and inhibitory inputs to BFCN$_{\rightarrow ACA}$ neurons.

Table S6. Data summary for the whole-brain distribution of excitatory and inhibitory inputs to BFCN$_{\rightarrow BLA}$ neurons.

Table S7. Sex and age of animals used in this study.

SUPPLEMENTARY MOVIE LEGENDS

Movie S1. Distributions of retrogradely-labeled BFCN$_{\rightarrow ACA}$ and BFCN$_{\rightarrow BLA}$ neurons.

Movie S2. Whole-brain distributions of axons from BFCN$_{\rightarrow ACA}$ and BFCN$_{\rightarrow BLA}$ neurons.

Movie S3. Whole-brain distributions of RV-labeled *Vglut*+ and *Vgat*+ neurons presynaptic to BFCN$_{\rightarrow ACA}$ neurons.

Movie S4. Whole-brain distributions of RV-labeled *Vglut*+ and *Vgat*+ neurons presynaptic to BFCN$_{\rightarrow BLA}$ neurons.
